# Supplementary material for: Potential impact, costs, and benefits of population-wide screening interventions for tuberculosis in Viet Nam: A mathematical modelling study
Source: PLOS Glob Public Health. 2025 Sep 10;5(9):e0005050. doi: 10.1371/journal.pgph.0005050 (PMC12422431; doi:10.1371/journal.pgph.0005050)
Supplement: S1 Text — (PDF) [file pgph.0005050.s001.pdf]

Potential impact, costs, and benefits of population-wide screening interventions for tuberculosis in Viet Nam: a mathematical modelling study

Alvaro Schwalb[1,2,3], Katherine C. Horton[1,2], Jon C. Emery[1,2], Martin J. Harker[1,2,4], Lara Goscé[1,2], Lara D. Veeken[5], Frances L. Garden[6,7], Hai Viet Nguyen[8], Thu-Anh Nguyen[9,10,11,12], Khanh Luu Boi[12], Frank Cobelens[13,14], Greg J. Fox[10,11,12], Van Luong Dinh[15,16], Hoa Binh Nguyen[15,16], Guy B. Marks[6,12,17,18], Rein M.G.J. Houben[1,2]

Affiliations:

1. TB Modelling Group, TB Centre, London School of Hygiene and Tropical Medicine, London, United Kingdom; 2. Department of Infectious Disease Epidemiology, London School of Hygiene and Tropical Medicine, London, United Kingdom; 3. Instituto de Medicina Tropical Alexander von Humboldt, Universidad Peruana Cayetano Heredia, Lima, Peru; 4. Global Health Economics Centre, London School of Hygiene and Tropical Medicine, London, United Kingdom; 5. Department of Internal Medicine and Radboud Community for Infectious Diseases, Radboud University Medical Center, Nijmegen, the Netherlands; 6. South West Sydney Clinical Campuses, University of New South Wales, Sydney, Australia; 7. Ingham Institute of Applied Medical Research, Sydney, Australia; 8. Ministry of Health, Hanoi, Viet Nam; 9. The University of Sydney Vietnam Institute, Ho Chi Minh City, Viet Nam; 10. Faculty of Medicine and Health, University of Sydney, Sydney, Australia; 11. The University of Sydney Institute for Infectious Diseases, Sydney, Australia; 12. Woolcock Institute of Medical Research, Sydney, Australia; 13. Department of Global Health, Amsterdam University Medical Centers, University of Amsterdam, Amsterdam, the Netherlands; 14. Amsterdam Institute for Global Health and Development, Amsterdam, the Netherlands; 15. National Lung Hospital, National Tuberculosis Control Programme, Hanoi, Viet Nam; 16. Hanoi Medical University, Hanoi, Viet Nam; 17. School of Clinical Medicine, University of New South Wales, Sydney, Australia; 18. Burnet Institute, Melbourne, Australia.

Corresponding author: A. Schwalb, London School of Hygiene & Tropical Medicine, Keppel Street, London WC1E 7HT, UK (alvaro.schwalb@lshtm.ac.uk)

S1 Text. Baseline model structure

We developed a compartmental model of tuberculosis (TB) natural history, adapting features of previously published models [1]. The model structure is shown in **Figure 1** on the manuscript. Model parameters used and their definitions are provided in **S2 Table** in a later section within this document. This model was run from 1500 to 2020 with some time-varying parameters. The model tracked a closed population of 100,000 adults (≥15 years old).

We represented TB natural history with nine distinct compartments allowing for *Mycobacterium tuberculosis* (*Mtb*) infection through an annual risk of infection (ARI). Disease state classification was informed by the ICE-TB framework, and naming follows current World Health Organization (WHO) definitions [2,3]:

- Non-infectious TB disease (nTB): individuals with inflammatory pathology (evidenced through imaging methods) in the absence of bacteriological evidence of TB disease and infectiousness, regardless of symptoms of active TB disease.
- Asymptomatic TB disease (aTB): individuals with bacteriological evidence of TB disease, such as a positive sputum smear, nucleic acid amplification test (NAAT), or culture, who do not report symptoms of active TB disease on screening.
- Symptomatic TB disease (sTB): individuals with bacteriological evidence of TB disease with symptoms of active TB disease.
- Infectious disease: refers to bacteriologically positive disease; as such, it includes asymptomatic and symptomatic disease.
- TB disease: Any state of non-infectious, asymptomatic, or symptomatic disease.

The ARI λ depends upon the contact parameter β and the prevalence of infectious disease (i.e., asymptomatic and symptomatic TB). Additionally, the relative infectiousness κ of asymptomatic TB is also considered. The formula for the ARI λ is presented later in this document. Individuals in the *Susceptible* (S) compartment could become infected with *Mtb* and progress to the *Infection* (I) compartment. From *Infection* (I), three pathways are possible: (i) self-clearance of infection (i.e. *Cleared* (C)) at rate *infcle*, (ii) progression to *Non-infectious* (nTB) at rate *infnon*, and (iii) progression to *Asymptomatic* (aTB) at rate *infasy*. TB transmission in the model (i.e., transition into the *Infected* (I) compartment) can occur via the ARI λ through four distinct routes: through first infection from *Susceptible* (S), through reinfection after self-clearance from *Cleared* (C), through reinfection after self-cure from *Recovered* (R) accounting for protection from reinfection π, and through reinfection after treatment from *Treated* (Tr)

accounting for increased risk of reinfection ρ. TB disease states are sequentially depicted in the model in the *Non-infectious* (nTB), *Asymptomatic* (aTB), and *Symptomatic* (sTB) compartments, allowing progression (denoted with parameters *nonasy* and *asysym*) and regression (denoted with parameters *asynon* and *symasy*). For the *Non-infectious* (nTB) compartment, individuals can transition out of disease states by self-cure into the *Recovered* (R) compartment at rate *nonrec*; here, reinfection can occur via the ARI but we assume there is protection from reinfection π. The model assumes that TB diagnosis and treatment only occur for individuals in the *Symptomatic* (sTB) compartment at rate θ. Furthermore, it accounts for TB-specific mortality μ_{TB} in this compartment. Once in *Treatment* (Tx), there can be treatment failure at rate ϕ and treatment completion at rate δ. Finally, in the *Treated* (Tr) compartment, reinfection can occur via the ARI λ parameter.

The model also accounts for background mortality having a fixed rate μ (representing an age expectancy of 70 years) in each compartment. As a closed population model, the sum of all background and TB-specific mortality is fed back into the *Susceptible* compartment through the ω parameter.

References

1. Horton KC, Richards AS, Emery JC, Esmail H, Houben RMGJ. Reevaluating progression and pathways following *Mycobacterium tuberculosis* infection within the spectrum of tuberculosis. Proc Natl Acad Sci U S A. 2023;120: e2221186120. doi:10.1073/pnas.2221186120

2. Coussens AK, Zaidi SMA, Allwood BW, Dewan PK, Gray G, Kohli M, et al. Classification of early tuberculosis states to guide research for improved care and prevention: an international Delphi consensus exercise. Lancet Respir Med. 2024. doi:10.1016/S2213-2600(24)00028-6

3. Falzon D, Miller C, Law I, Floyd K, Arinaminpathy N, Zignol M, et al. Managing tuberculosis before the onset of symptoms. Lancet Respir Med. 2024;0. doi:10.1016/s2213-2600(24)00372-2
