# Supplementary material for: Potential impact, costs, and benefits of population-wide screening interventions for tuberculosis in Viet Nam: A mathematical modelling study
Source: PLOS Glob Public Health. 2025 Sep 10;5(9):e0005050. doi: 10.1371/journal.pgph.0005050 (PMC12422431; doi:10.1371/journal.pgph.0005050)
Supplement: S2 Text — (PDF) [file pgph.0005050.s002.pdf]

Potential impact, costs, and benefits of population-wide screening interventions for tuberculosis in Viet Nam: a mathematical modelling study

Alvaro Schwalb[1,2,3], Katherine C. Horton[1,2], Jon C. Emery[1,2], Martin J. Harker[1,2,4], Lara Goscé[1,2], Lara D. Veeken[5], Frances L. Garden[6,7], Hai Viet Nguyen[8], Thu-Anh Nguyen[9,10,11,12], Khanh Luu Boi[12], Frank Cobelens[13,14], Greg J. Fox[10,11,12], Van Luong Dinh[15,16], Hoa Binh Nguyen[15,16], Guy B. Marks[6,12,17,18], Rein M.G.J. Houben[1,2]

Affiliations:

1. TB Modelling Group, TB Centre, London School of Hygiene and Tropical Medicine, London, United Kingdom; 2. Department of Infectious Disease Epidemiology, London School of Hygiene and Tropical Medicine, London, United Kingdom; 3. Instituto de Medicina Tropical Alexander von Humboldt, Universidad Peruana Cayetano Heredia, Lima, Peru; 4. Global Health Economics Centre, London School of Hygiene and Tropical Medicine, London, United Kingdom; 5. Department of Internal Medicine and Radboud Community for Infectious Diseases, Radboud University Medical Center, Nijmegen, the Netherlands; 6. South West Sydney Clinical Campuses, University of New South Wales, Sydney, Australia; 7. Ingham Institute of Applied Medical Research, Sydney, Australia; 8. Ministry of Health, Hanoi, Viet Nam; 9. The University of Sydney Vietnam Institute, Ho Chi Minh City, Viet Nam; 10. Faculty of Medicine and Health, University of Sydney, Sydney, Australia; 11. The University of Sydney Institute for Infectious Diseases, Sydney, Australia; 12. Woolcock Institute of Medical Research, Sydney, Australia; 13. Department of Global Health, Amsterdam University Medical Centers, University of Amsterdam, Amsterdam, the Netherlands; 14. Amsterdam Institute for Global Health and Development, Amsterdam, the Netherlands; 15. National Lung Hospital, National Tuberculosis Control Programme, Hanoi, Viet Nam; 16. Hanoi Medical University, Hanoi, Viet Nam; 17. School of Clinical Medicine, University of New South Wales, Sydney, Australia; 18. Burnet Institute, Melbourne, Australia.

Corresponding author: A. Schwalb, London School of Hygiene & Tropical Medicine, Keppel Street, London WC1E 7HT, UK (alvaro.schwalb@lshtm.ac.uk)

S2 Text. Baseline model equations

A series of ordinary differential equations were set in place to represent the model structure mathematically. Parameter symbols and descriptions are outlined in **S2 Table**. Parameter names indicate direction (i.e., *infcle* denotes from *Infection* to *Cleared*), and subscript *t* denotes parameters that vary over time. All nine compartments are represented: *Susceptible* (S), *Infected* (I), *Cleared* (C), *Recovered* (R), *Non-infectious* (nTB), *Asymptomatic* (aTB), *Symptomatic* (sTB), *Treatment* (Tx), *Treated* (Tr). Given N = 100,000, then:

$$\frac{dS}{dt} = \mu \cdot (N - S) + \mu_{TB,t} \cdot sTB - \lambda \cdot S$$

$$\frac{dI}{dt} = \lambda \cdot (S + C + \pi \cdot R + \rho \cdot Tr) - I \cdot (infcle + infnon + infasy + \mu)$$

$$\frac{dC}{dt} = infcle \cdot I - C \cdot (\lambda + \mu)$$

$$\frac{dR}{dt} = nonrec \cdot nTB - R \cdot (\lambda \cdot \pi + \mu)$$

$$\frac{dnTB}{dt} = infnon \cdot I + asynon \cdot aTB - nTB \cdot (nonrec + nonasy + \mu)$$

$$\frac{daTB}{dt} = infasy \cdot I + nonasy \cdot nTB + symasy \cdot sTB - aTB \cdot (asynon + asysym + \mu)$$

$$\frac{dsTB}{dt} = asysym \cdot aTB - sTB \cdot \left(symasy + \theta_t + \mu_{TB,t} + \mu\right) + \varphi_t \cdot Tx$$

$$\frac{dTx}{dt} = \theta_t \cdot sTB - Tx \cdot (\varphi_t + \delta + \mu)$$

$$\frac{dTr}{dt} = \delta \cdot Tx - Tr \cdot (\lambda \cdot \rho + \mu)$$

As mentioned before, the ARI λ depends upon the contact parameter β and the prevalence of infectious disease (i.e., asymptomatic and symptomatic TB) and its equation is expressed below.

$$\lambda = \frac{\beta \cdot (\kappa \cdot aTB + sTB)}{N}$$
