# Supplementary material for: Potential impact, costs, and benefits of population-wide screening interventions for tuberculosis in Viet Nam: A mathematical modelling study
Source: PLOS Glob Public Health. 2025 Sep 10;5(9):e0005050. doi: 10.1371/journal.pgph.0005050 (PMC12422431; doi:10.1371/journal.pgph.0005050)
Supplement: S3 Text — (PDF) [file pgph.0005050.s003.pdf]

Potential impact, costs, and benefits of population-wide screening interventions for tuberculosis in Viet Nam: a mathematical modelling study

Alvaro Schwalb[1,2,3], Katherine C. Horton[1,2], Jon C. Emery[1,2], Martin J. Harker[1,2,4], Lara Goscé[1,2], Lara D. Veeken[5], Frances L. Garden[6,7], Hai Viet Nguyen[8], Thu-Anh Nguyen[9,10,11,12], Khanh Luu Boi[12], Frank Cobelens[13,14], Greg J. Fox[10,11,12], Van Luong Dinh[15,16], Hoa Binh Nguyen[15,16], Guy B. Marks[6,12,17,18], Rein M.G.J. Houben[1,2]

Affiliations:

1. TB Modelling Group, TB Centre, London School of Hygiene and Tropical Medicine, London, United Kingdom; 2. Department of Infectious Disease Epidemiology, London School of Hygiene and Tropical Medicine, London, United Kingdom; 3. Instituto de Medicina Tropical Alexander von Humboldt, Universidad Peruana Cayetano Heredia, Lima, Peru; 4. Global Health Economics Centre, London School of Hygiene and Tropical Medicine, London, United Kingdom; 5. Department of Internal Medicine and Radboud Community for Infectious Diseases, Radboud University Medical Center, Nijmegen, the Netherlands; 6. South West Sydney Clinical Campuses, University of New South Wales, Sydney, Australia; 7. Ingham Institute of Applied Medical Research, Sydney, Australia; 8. Ministry of Health, Hanoi, Viet Nam; 9. The University of Sydney Vietnam Institute, Ho Chi Minh City, Viet Nam; 10. Faculty of Medicine and Health, University of Sydney, Sydney, Australia; 11. The University of Sydney Institute for Infectious Diseases, Sydney, Australia; 12. Woolcock Institute of Medical Research, Sydney, Australia; 13. Department of Global Health, Amsterdam University Medical Centers, University of Amsterdam, Amsterdam, the Netherlands; 14. Amsterdam Institute for Global Health and Development, Amsterdam, the Netherlands; 15. National Lung Hospital, National Tuberculosis Control Programme, Hanoi, Viet Nam; 16. Hanoi Medical University, Hanoi, Viet Nam; 17. School of Clinical Medicine, University of New South Wales, Sydney, Australia; 18. Burnet Institute, Melbourne, Australia.

Corresponding author: A. Schwalb, London School of Hygiene & Tropical Medicine, Keppel Street, London WC1E 7HT, UK (alvaro.schwalb@lshtm.ac.uk)

S3 Text. Calibration methodology

We calibrated the model using history matching with emulation, a calibration method that explores high-dimensional parameter spaces efficiently [1]. History matching refers to the exploration of the ranges of parameters given and identifying parameter sets that give rise to model outputs that match empirical data [1]. History matching progresses through multiple iterations (referred to as waves), where implausible areas of parameters (i.e., values where no match is found) are identified and discarded [1]. This process is made efficient with the use of emulators, which provide approximations of model outputs orders of magnitudes faster than the model [1]. As a result of multiple waves, the implausible space is reduced, resulting in parameter sets that match calibration targets.

History matching with emulation was implemented using the *hmer* package in R [2]. Calibration targets were TB epidemiological and demographic data of Viet Nam (**S1 Table**). The model comprised 23 dynamic parameters which are described in **S2 Table**. The parameter ranges (priors) and sources are outlined. The non-implausible points (posteriors) were calculated as the median and corresponding 95% uncertainty intervals, calculated as the 2.5th to 97.5th percentiles of the parameter sets.

References

1. Scarponi D, Iskauskas A, Clark RA, Vernon I, McKinley TJ, Goldstein M, et al. Demonstrating multi-country calibration of a tuberculosis model using new history matching and emulation package - hmer. Epidemics. 2023;43: 100678. doi:10.1016/j.epidem.2023.100678

2. Iskauskas A, McKinley TJ. hmer: history matching and emulation package. In: The Comprehensive R Archive Network [Internet]. 2022. Available: https://CRAN.R-project.org/package=hmer
