# Supplementary material for: Potential impact, costs, and benefits of population-wide screening interventions for tuberculosis in Viet Nam: A mathematical modelling study
Source: PLOS Glob Public Health. 2025 Sep 10;5(9):e0005050. doi: 10.1371/journal.pgph.0005050 (PMC12422431; doi:10.1371/journal.pgph.0005050)
Supplement: S5 Text — (PDF) [file pgph.0005050.s005.pdf]

Potential impact, costs, and benefits of population-wide screening interventions for tuberculosis in Viet Nam: a mathematical modelling study

Alvaro Schwalb[1,2,3], Katherine C. Horton[1,2], Jon C. Emery[1,2], Martin J. Harker[1,2,4], Lara Goscé[1,2], Lara D. Veeken[5], Frances L. Garden[6,7], Hai Viet Nguyen[8], Thu-Anh Nguyen[9,10,11,12], Khanh Luu Boi[12], Frank Cobelens[13,14], Greg J. Fox[10,11,12], Van Luong Dinh[15,16], Hoa Binh Nguyen[15,16], Guy B. Marks[6,12,17,18], Rein M.G.J. Houben[1,2]

Affiliations:

1. TB Modelling Group, TB Centre, London School of Hygiene and Tropical Medicine, London, United Kingdom; 2. Department of Infectious Disease Epidemiology, London School of Hygiene and Tropical Medicine, London, United Kingdom; 3. Instituto de Medicina Tropical Alexander von Humboldt, Universidad Peruana Cayetano Heredia, Lima, Peru; 4. Global Health Economics Centre, London School of Hygiene and Tropical Medicine, London, United Kingdom; 5. Department of Internal Medicine and Radboud Community for Infectious Diseases, Radboud University Medical Center, Nijmegen, the Netherlands; 6. South West Sydney Clinical Campuses, University of New South Wales, Sydney, Australia; 7. Ingham Institute of Applied Medical Research, Sydney, Australia; 8. Ministry of Health, Hanoi, Viet Nam; 9. The University of Sydney Vietnam Institute, Ho Chi Minh City, Viet Nam; 10. Faculty of Medicine and Health, University of Sydney, Sydney, Australia; 11. The University of Sydney Institute for Infectious Diseases, Sydney, Australia; 12. Woolcock Institute of Medical Research, Sydney, Australia; 13. Department of Global Health, Amsterdam University Medical Centers, University of Amsterdam, Amsterdam, the Netherlands; 14. Amsterdam Institute for Global Health and Development, Amsterdam, the Netherlands; 15. National Lung Hospital, National Tuberculosis Control Programme, Hanoi, Viet Nam; 16. Hanoi Medical University, Hanoi, Viet Nam; 17. School of Clinical Medicine, University of New South Wales, Sydney, Australia; 18. Burnet Institute, Melbourne, Australia.

Corresponding author: A. Schwalb, London School of Hygiene & Tropical Medicine, Keppel Street, London WC1E 7HT, UK (alvaro.schwalb@lshtm.ac.uk)

S5 Text. Disability-adjusted life years calculations

Since our compartmental model does not track ageing, we opted to estimate mean lifetime disability-adjusted life years (DALYs) per incident TB. For this, we used the total DALYs for TB disease (0.40 million; 95%CI: 0.33-0.49) and post-TB (0.85 million; 95%CI: 0.57-1.22) in Viet Nam in 2019, as estimated by Menzies et al [1]. Then, considering the number of incident TB estimated in Viet Nam in 2019 (169,000), we calculated the point value for lifetime DALYs per incident TB: 2.4 (95%CI: 2.0-2.9) for TB disease and 5.0 (95%CI: 3.4-7.2) for post-TB [2,3].

To estimate the DALYs lived with post-TB, we used the weighted average age of individuals with TB in Viet Nam (49 years) from the WHO Global TB Report and obtained the life expectancy at that age (29.5 years) from the United Nations World Population Prospects [1,3]. Next, we estimated the proportion of an individual's remaining lifetime that would occur between the start of the population-wide screening interventions in 2025 and the time horizon of 2050, a period chosen to align with the duration of the implementation of the intervention and evaluation timeframe. Ultimately, lifetime DALYs per incident TB were calculated as the DALYs due to TB disease episode plus the DALYs lived with post-TB, discounted at a rate of 3% per year from 2025 [4].

References

1. United Nations. World Population Prospects - Population Division. In: World Population Prospects 2022 [Internet]. [cited Jun 2023]. Available: https://population.un.org/wpp/

2. Menzies NA, Quaife M, Allwood BW, Byrne AL, Coussens AK, Harries AD, et al. Lifetime burden of disease due to incident tuberculosis: a global reappraisal including post-tuberculosis sequelae. Lancet Glob Health. 2021;9: e1679–e1687. doi:10.1016/S2214-109X(21)00367-3

3. World Health Organization. Global Tuberculosis Report 2022. Geneva: WHO; 2022.

4. Wilkinson T, Sculpher MJ, Claxton K, Revill P, Briggs A, Cairns JA, et al. The International Decision Support Initiative reference case for economic evaluation: An aid to thought. Value Health. 2016;19: 921–928. doi:10.1016/j.jval.2016.04.015
