# Supplementary material for: Potential impact, costs, and benefits of population-wide screening interventions for tuberculosis in Viet Nam: A mathematical modelling study
Source: PLOS Glob Public Health. 2025 Sep 10;5(9):e0005050. doi: 10.1371/journal.pgph.0005050 (PMC12422431; doi:10.1371/journal.pgph.0005050)
Supplement: S1 Table — (PDF) [file pgph.0005050.s010.pdf]

Potential impact, costs, and benefits of population-wide screening interventions for tuberculosis in Viet Nam: a mathematical modelling study

Alvaro Schwalb[1,2,3], Katherine C. Horton[1,2], Jon C. Emery[1,2], Martin J. Harker[1,2,4], Lara Goscé[1,2], Lara D. Veeken[5], Frances L. Garden[6,7], Hai Viet Nguyen[8], Thu-Anh Nguyen[9,10,11,12], Khanh Luu Boi[12], Frank Cobelens[13,14], Greg J. Fox[10,11,12], Van Luong Dinh[15,16], Hoa Binh Nguyen[15,16], Guy B. Marks[6,12,17,18], Rein M.G.J. Houben[1,2]

Affiliations:

1. TB Modelling Group, TB Centre, London School of Hygiene and Tropical Medicine, London, United Kingdom; 2. Department of Infectious Disease Epidemiology, London School of Hygiene and Tropical Medicine, London, United Kingdom; 3. Instituto de Medicina Tropical Alexander von Humboldt, Universidad Peruana Cayetano Heredia, Lima, Peru; 4. Global Health Economics Centre, London School of Hygiene and Tropical Medicine, London, United Kingdom; 5. Department of Internal Medicine and Radboud Community for Infectious Diseases, Radboud University Medical Center, Nijmegen, the Netherlands; 6. South West Sydney Clinical Campuses, University of New South Wales, Sydney, Australia; 7. Ingham Institute of Applied Medical Research, Sydney, Australia; 8. Ministry of Health, Hanoi, Viet Nam; 9. The University of Sydney Vietnam Institute, Ho Chi Minh City, Viet Nam; 10. Faculty of Medicine and Health, University of Sydney, Sydney, Australia; 11. The University of Sydney Institute for Infectious Diseases, Sydney, Australia; 12. Woolcock Institute of Medical Research, Sydney, Australia; 13. Department of Global Health, Amsterdam University Medical Centers, University of Amsterdam, Amsterdam, the Netherlands; 14. Amsterdam Institute for Global Health and Development, Amsterdam, the Netherlands; 15. National Lung Hospital, National Tuberculosis Control Programme, Hanoi, Viet Nam; 16. Hanoi Medical University, Hanoi, Viet Nam; 17. School of Clinical Medicine, University of New South Wales, Sydney, Australia; 18. Burnet Institute, Melbourne, Australia.

Corresponding author: A. Schwalb, London School of Hygiene & Tropical Medicine, Keppel Street, London WC1E 7HT, UK (alvaro.schwalb@lshtm.ac.uk)

S1 Table. Calibration targets.

Target	Year	Value [95%CI]	Source
TB prevalence per 100,000 people	2007	250 [202 - 310]	[1]
	2018	227 [177 - 290]	[1]
TB mortality rate per 100,000 people	2000	59.7 [37.3 - 87.7]	[2,3]
	2010	33.5 [22.8 - 45.7]	[2,3]
TB notification rate per 100,000 people	2010	79.4 [63.5 - 95.3]	[2,3]
	2020	73.2 [58.5 - 87.8]	[2,3]
Proportion asymptomatic TB	2007	0.70 [0.56 - 0.84]	[4]
	2018	0.66 [0.53 - 0.79]	[4]

Calibration targets for TB epidemiology in Viet Nam, with brackets indicating 95% confidence intervals (95%CI), were set for the adult population aged 15 years and older. TB prevalence refers specifically to infectious TB (i.e., asymptomatic and symptomatic); TB mortality reflects deaths from symptomatic TB; TB notification corresponds to the number of individuals with symptomatic TB initiating treatment through the business-as-usual approach; and the proportion of asymptomatic TB denotes the share of all infectious TB that is asymptomatic. Estimates for the TB prevalence used as calibration targets differ from those available in reference; since publication, an observed disparity in the proportional decline was corrected by the authors, and the estimates provided here reflect this correction.

1

References

1. Nguyen HV, Nguyen HB, Nguyen NV, Cobelens F, Finlay A, Dao CH, et al. Decline of Tuberculosis Burden in Vietnam Measured by Consecutive National Surveys, 2007-2017. Emerg Infect Dis. 2021;27: 872–879. doi:10.3201/eid2703.204253

2. World Health Organization. Global Tuberculosis Report 2022. Geneva: WHO; 2022.

3. United Nations. World Population Prospects - Population Division. In: World Population Prospects 2022 [Internet]. [cited Jun 2023]. Available: https://population.un.org/wpp/

4. Emery JC, Dodd PJ, Banu S, Frascella B, Garden FL, Horton KC, et al. Estimating the contribution of subclinical tuberculosis disease to transmission: An individual patient data analysis from prevalence surveys. Elife. 2023;12. doi:10.7554/eLife.82469
