# Supplementary material for: Potential impact, costs, and benefits of population-wide screening interventions for tuberculosis in Viet Nam: A mathematical modelling study
Source: PLOS Glob Public Health. 2025 Sep 10;5(9):e0005050. doi: 10.1371/journal.pgph.0005050 (PMC12422431; doi:10.1371/journal.pgph.0005050)
Supplement: S2 Table — (PDF) [file pgph.0005050.s011.pdf]

Potential impact, costs, and benefits of population-wide screening interventions for tuberculosis in Viet Nam: a mathematical modelling study

Alvaro Schwalb[1,2,3], Katherine C. Horton[1,2], Jon C. Emery[1,2], Martin J. Harker[1,2,4], Lara Goscé[1,2], Lara D. Veeken[5], Frances L. Garden[6,7], Hai Viet Nguyen[8], Thu-Anh Nguyen[9,10,11,12], Khanh Luu Boi[12], Frank Cobelens[13,14], Greg J. Fox[10,11,12], Van Luong Dinh[15,16], Hoa Binh Nguyen[15,16], Guy B. Marks[6,12,17,18], Rein M.G.J. Houben[1,2]

Affiliations:

1. TB Modelling Group, TB Centre, London School of Hygiene and Tropical Medicine, London, United Kingdom; 2. Department of Infectious Disease Epidemiology, London School of Hygiene and Tropical Medicine, London, United Kingdom; 3. Instituto de Medicina Tropical Alexander von Humboldt, Universidad Peruana Cayetano Heredia, Lima, Peru; 4. Global Health Economics Centre, London School of Hygiene and Tropical Medicine, London, United Kingdom; 5. Department of Internal Medicine and Radboud Community for Infectious Diseases, Radboud University Medical Center, Nijmegen, the Netherlands; 6. South West Sydney Clinical Campuses, University of New South Wales, Sydney, Australia; 7. Ingham Institute of Applied Medical Research, Sydney, Australia; 8. Ministry of Health, Hanoi, Viet Nam; 9. The University of Sydney Vietnam Institute, Ho Chi Minh City, Viet Nam; 10. Faculty of Medicine and Health, University of Sydney, Sydney, Australia; 11. The University of Sydney Institute for Infectious Diseases, Sydney, Australia; 12. Woolcock Institute of Medical Research, Sydney, Australia; 13. Department of Global Health, Amsterdam University Medical Centers, University of Amsterdam, Amsterdam, the Netherlands; 14. Amsterdam Institute for Global Health and Development, Amsterdam, the Netherlands; 15. National Lung Hospital, National Tuberculosis Control Programme, Hanoi, Viet Nam; 16. Hanoi Medical University, Hanoi, Viet Nam; 17. School of Clinical Medicine, University of New South Wales, Sydney, Australia; 18. Burnet Institute, Melbourne, Australia.

Corresponding author: A. Schwalb, London School of Hygiene & Tropical Medicine, Keppel Street, London WC1E 7HT, UK (alvaro.schwalb@lshtm.ac.uk)

S2 Table. Model parameter description, ranges, and non-implausible points.

Parameters	Description	Ranges	Non-implausible ranges [95%UI]	Sources
beta (β)	Transmission coefficient	6.00 - 20.00	14.16 [8.82 - 19.29]	-
kappa (κ)	Relative transmission from asymptomatic TB	0.62 - 1.00	0.82 [0.64 - 0.98]	[1]
pi (π)	Relative risk of reinfection after recovery from non-infectious TB	0.14 - 0.30	0.21 [0.15 - 0.29]	[2]
rho (ρ)	Relative risk of reinfection after treatment completion	2.14 - 4.27	3.15 [2.23 - 4.19]	[3]
infcle	Rate of clearance from infection per year	0.93 - 3.30	1.90 [1.09 - 2.94]	[4]
infnon	Rate of progression from infection to non-infectious TB per year	0.04 - 0.23	0.16 [0.06 - 0.22]	[4]
infasy	Rate of progression from infection to asymptomatic TB per year	0.01 - 0.10	0.06 [0.01 - 0.10]	[4]
nonrec	Rate of recovery from non-infectious TB per year	0.14 - 0.23	0.18 [0.14 - 0.22]	[4]
nonasy	Rate of progression from non-infectious to asymptomatic TB per year	0.21 - 0.28	0.25 [0.21 - 0.28]	[4]
asynon	Rate of recovery from asymptomatic to non-infectious TB per year	1.24 - 2.03	1.66 [1.30 - 1.99]	[4]
asysym	Rate of progression from asymptomatic to symptomatic TB per year	0.56 - 0.94	0.88 [0.76 - 0.94]	[4]
symasy	Rate of recovery from symptomatic to asymptomatic TB per year	0.46 - 0.72	0.54 [0.47 - 0.68]	[4]
theta_ini (θ_t)	Rate of treatment initiation from symptomatic TB per year (initial)	0.00 - 0.57	0.46 [0.34 - 0.56]	-
theta_fin (θ_t)	Rate of treatment initiation from symptomatic TB per year (final)	0.57 - 0.77	0.71 [0.60 - 0.76]	[5]
delta (δ)	Rate of treatment completion per year	2.00	-	[6]
phi_ini (ϕ_t)	Rate of treatment failure per year (initial)	0.11 - 1.00	0.63 [0.21 - 0.97]	-
phi_fin (ϕ_t)	Rate of treatment failure per year (final)	0.07 - 0.11	0.09 [0.07 - 0.11]	[5]

mutb_ini ($\mu_{TB,t}$)	TB-specific mortality rate per year (initial)	0.28 - 0.38	0.34 [0.29 - 0.37]	[7]
mutb_fin ($\mu_{TB,t}$)	TB-specific mortality rate per year (final)	0.00 - 0.28	0.17 [0.07 - 0.27]	-
mu (μ)	Background mortality rate per year	0.014	-	-

Description of model parameters for the deterministic TB transmission model calibrated to TB epidemiology in Viet Nam. The table presents prior ranges and the posterior median values with corresponding 95% uncertainty intervals (95%UIs) for non-implausible ranges. Constant values are shown where ranges are not applicable. Some parameters are labelled as “initial” and “final”; the initial value remains constant from 1500 to 1999 and is then scaled linearly (up or down) to reach the final value by 2020. All parameters are expressed per year.

References

1. Emery JC, Dodd PJ, Banu S, Frascella B, Garden FL, Horton KC, et al. Estimating the contribution of subclinical tuberculosis disease to transmission: An individual patient data analysis from prevalence surveys. Elife. 2023;12. doi:10.7554/eLife.82469

2. Andrews JR, Noubary F, Walensky RP, Cerda R, Losina E, Horsburgh CR. Risk of progression to active tuberculosis following reinfection with Mycobacterium tuberculosis. Clin Infect Dis. 2012;54: 784–791. doi:10.1093/cid/cir951

3. Verver S, Warren RM, Beyers N, Richardson M, van der Spuy GD, Borgdorff MW, et al. Rate of reinfection tuberculosis after successful treatment is higher than rate of new tuberculosis. Am J Respir Crit Care Med. 2005;171: 1430–1435. doi:10.1164/rccm.200409-1200OC

4. Horton KC, Richards AS, Emery JC, Esmail H, Houben RMGJ. Reevaluating progression and pathways following *Mycobacterium tuberculosis* infection within the spectrum of tuberculosis. Proc Natl Acad Sci U S A. 2023;120: e2221186120. doi:10.1073/pnas.2221186120

5. World Health Organization. Global Tuberculosis Report 2022. Geneva: WHO; 2022.

6. World Health Organization. WHO Consolidated Guidelines on Tuberculosis. Module 4: Treatment - Drug-susceptible tuberculosis treatment. Geneva, Switzerland: WHO; 2022.

7. Richards AS, Sossen B, Emery JC, Horton KC, Heinsohn T, Frascella B, et al. Quantifying progression and regression across the spectrum of pulmonary tuberculosis: a data synthesis study. Lancet Glob Health. 2023;11: e684–e692. doi:10.1016/S2214-109X(23)00082-7
