# Supplementary material for: Potential impact, costs, and benefits of population-wide screening interventions for tuberculosis in Viet Nam: A mathematical modelling study
Source: PLOS Glob Public Health. 2025 Sep 10;5(9):e0005050. doi: 10.1371/journal.pgph.0005050 (PMC12422431; doi:10.1371/journal.pgph.0005050)
Supplement: S3 Table — (PDF) [file pgph.0005050.s012.pdf]

Potential impact, costs, and benefits of population-wide screening interventions for tuberculosis in Viet Nam: a mathematical modelling study

Alvaro Schwalb[1,2,3], Katherine C. Horton[1,2], Jon C. Emery[1,2], Martin J. Harker[1,2,4], Lara Goscé[1,2], Lara D. Veeken[5], Frances L. Garden[6,7], Hai Viet Nguyen[8], Thu-Anh Nguyen[9,10,11,12], Khanh Luu Boi[12], Frank Cobelens[13,14], Greg J. Fox[10,11,12], Van Luong Dinh[15,16], Hoa Binh Nguyen[15,16], Guy B. Marks[6,12,17,18], Rein M.G.J. Houben[1,2]

Affiliations:

1. TB Modelling Group, TB Centre, London School of Hygiene and Tropical Medicine, London, United Kingdom; 2. Department of Infectious Disease Epidemiology, London School of Hygiene and Tropical Medicine, London, United Kingdom; 3. Instituto de Medicina Tropical Alexander von Humboldt, Universidad Peruana Cayetano Heredia, Lima, Peru; 4. Global Health Economics Centre, London School of Hygiene and Tropical Medicine, London, United Kingdom; 5. Department of Internal Medicine and Radboud Community for Infectious Diseases, Radboud University Medical Center, Nijmegen, the Netherlands; 6. South West Sydney Clinical Campuses, University of New South Wales, Sydney, Australia; 7. Ingham Institute of Applied Medical Research, Sydney, Australia; 8. Ministry of Health, Hanoi, Viet Nam; 9. The University of Sydney Vietnam Institute, Ho Chi Minh City, Viet Nam; 10. Faculty of Medicine and Health, University of Sydney, Sydney, Australia; 11. The University of Sydney Institute for Infectious Diseases, Sydney, Australia; 12. Woolcock Institute of Medical Research, Sydney, Australia; 13. Department of Global Health, Amsterdam University Medical Centers, University of Amsterdam, Amsterdam, the Netherlands; 14. Amsterdam Institute for Global Health and Development, Amsterdam, the Netherlands; 15. National Lung Hospital, National Tuberculosis Control Programme, Hanoi, Viet Nam; 16. Hanoi Medical University, Hanoi, Viet Nam; 17. School of Clinical Medicine, University of New South Wales, Sydney, Australia; 18. Burnet Institute, Melbourne, Australia.

Corresponding author: A. Schwalb, London School of Hygiene & Tropical Medicine, Keppel Street, London WC1E 7HT, UK (alvaro.schwalb@lshtm.ac.uk)

S3 Table. Probability of a positive test per model state for each screening tool.

Test	State	Value (Range)	Description
Nucleic acid amplification test (NAAT, Xpert MTB/RIF Ultra)	Susceptible	0.006 (0.005 - 0.008)	(1 - specificity) for individuals in a community in Kampala, Uganda [1]
	Infected	0.006 (0.005 - 0.008)	(1 - specificity) for individuals in a community in Kampala, Uganda [1]
	Cleared	0.006 (0.005 - 0.008)	(1 - specificity) for individuals in a community in Kampala, Uganda [1]
	Recovered	0.040 (0.020 - 0.060)	(1 - specificity) for individuals screened positive for symptoms and/or CXR with a history of TB in the community [2]
	Non-infectious TB	0.044 (0.026 - 0.070)	(1 - specificity) for pulmonary TB from individuals in primary care facilities and local hospitals [3]
	Asymptomatic TB	0.775 (0.676 - 0.856)	Sensitivity for smear-negative TB from individuals in primary care facilities and local hospitals [3]
	Symptomatic TB	0.909 (0.862 - 0.947)	Sensitivity for pulmonary TB from individuals in primary care facilities and local hospitals [3]
	Treated	0.040 (0.020 - 0.060)	(1 - specificity) for individuals screened positive for symptoms and/or CXR with a history of TB in the community [2]
Chest radiography with CAD software interpretation (CXR)	Susceptible	0.085 (0.069 - 0.134)	Median and interquartile range for the proportion of national TB prevalence survey participants with abnormal CXR, regardless of TB status [4]
	Infected	0.085 (0.069 - 0.134)	Median and interquartile range for the proportion of national TB prevalence survey participants with abnormal CXR, regardless of TB status [4]
	Cleared	0.085 (0.069 - 0.134)	Median and interquartile range for the proportion of national TB prevalence survey participants with abnormal CXR, regardless of TB status [4]
	Recovered	0.503 (0.481 - 0.524)	Proportion with abnormal CXR suggestive of TB among participants of national TB prevalence survey reporting TB history [5]
	Non-infectious TB	0.677* (0.626 - 0.712)	Midpoint between the median and bounds of values for Recovered/Treated and Symptomatic TB [Assumption]
	Asymptomatic TB	0.677* (0.626 - 0.712)	Midpoint between the median and bounds of values for Recovered/Treated and Symptomatic TB [Assumption]
	Symptomatic TB	0.910 (0.900 - 0.920)	Sensitivity of CAD software for bacteriologically confirmed TB in screening use case [6]
	Treated	0.503 (0.481 - 0.524)	Proportion with abnormal CXR suggestive of TB among participants of national TB prevalence survey reporting TB history [5]

**Nucleic acid amplification test (NAAT, Xpert MTB/RIF)**	Susceptible	0.0022 (0.0016 - 0.0029)	(1 - specificity) for individuals in a community in selected villages in Ca Mau province, Viet Nam [7]
	Infected	0.0022 (0.0016 - 0.0029)	(1 - specificity) for individuals in a community in selected villages in Ca Mau province, Viet Nam [7]
	Cleared	0.0022 (0.0016 - 0.0029)	(1 - specificity) for individuals in a community in selected villages in Ca Mau province, Viet Nam [7]
	Recovered	0.026 (0.005 - 0.083)	(1 - specificity) for individuals with a history of TB in primary care facilities and local hospitals [3]
	Non-infectious TB	0.016 (0.007 - 0.030)	(1 - specificity) for pulmonary TB from individuals in primary care facilities and local hospitals [3]
	Asymptomatic TB	0.606 (0.484 - 0.717)	Sensitivity for smear-negative TB from individuals in primary care facilities and local hospitals [3]
	Symptomatic TB	0.847 (0.786 - 0.899)	Sensitivity for pulmonary TB from individuals in primary care facilities and local hospitals [3]
	Treated	0.026 (0.005 - 0.083)	(1 - specificity) for individuals with a history of TB in primary care facilities and local hospitals [3]
**Further investigation (informed by prolonged cough)**	Susceptible	0.061 (0.047 - 0.074)	Median and interquartile range for the proportion of national TB prevalence survey participants reporting prolonged cough, regardless of CXR or TB status [4]
	Infected	0.061 (0.047 - 0.074)	Median and interquartile range for the proportion of national TB prevalence survey participants reporting prolonged cough, regardless of CXR or TB status [4]
	Cleared	0.061 (0.047 - 0.074)	Median and interquartile range for the proportion of national TB prevalence survey participants reporting prolonged cough, regardless of CXR or TB status [4]
	Recovered	0.131 (0.089 - 0.162)	Midpoint between the median and bounds of values for S/I/C and Non-infectious TB [Assumption]
	Non-infectious TB	0.201 (0.129 - 0.249)	Median and interquartile range for the proportion of national TB prevalence survey participants reporting prolonged cough with abnormal CXR, regardless of TB status [4]
	Asymptomatic TB	1.00	Assuming a strong clinical appraisal that is able to diagnose confirmed TB [Assumption]
	Symptomatic TB	1.00	Assuming a strong clinical appraisal that is able to diagnose confirmed TB [Assumption]
	Treated	0.566 (0.545 - 0.581)	Midpoint between the median and bounds of values for Recovered and Asymptomatic/Symptomatic TB [Assumption]

Probability of a positive test result for each screening diagnostic tool by model state and were independently sampled from uniform distributions for each model run. For *Non-infectious* TB, the NAAT test positivity was informed by data from presumptive TB populations, given partial overlap in symptomatic individuals. Test positivity under further investigation refers to individuals who have tested positive based on a given screening algorithm. CXR positivity values do not correspond to a specific CAD

threshold. *In the sensitivity analysis evaluating revised CXR sensitivity, test positivity for *Non-infectious* and *Asymptomatic* TB was set to match that of the *Recovered* and *Treated* states. CAD: Computer-aided diagnosis; TB: Tuberculosis.

References

1. Kendall EA, Kitonsa PJ, Nalutaaya A, Erisa KC, Mukiibi J, Nakasolya O, et al. The Spectrum of Tuberculosis Disease in an Urban Ugandan Community and Its Health Facilities. Clin Infect Dis. 2021;72: e1035–e1043. doi:10.1093/cid/ciaa1824

2. World Health Organization. National tuberculosis prevalence surveys: what diagnostic algorithms should be used in future? Geneva, Switzerland: WHO; 2023 May. Available: https://iris.who.int/bitstream/handle/10665/367909/9789240073913-eng.pdf?sequence=1

3. Zifodya JS, Kreniske JS, Schiller I, Kohli M, Dendukuri N, Schumacher SG, et al. Xpert Ultra versus Xpert MTB/RIF for pulmonary tuberculosis and rifampicin resistance in adults with presumptive pulmonary tuberculosis. Cochrane Database Syst Rev. 2021;2: CD009593. doi:10.1002/14651858.CD009593.pub5

4. Frascella B, Richards AS, Sossen B, Emery JC, Odone A, Law I, et al. Subclinical tuberculosis disease - a review and analysis of prevalence surveys to inform definitions, burden, associations and screening methodology. Clin Infect Dis. 2020. doi:10.1093/cid/ciaa1402

5. Mungai B, Ong'angò J, Ku CC, Henrion MYR, Morton B, Joekes E, et al. Accuracy of computer-aided chest X-ray in community-based tuberculosis screening: Lessons from the 2016 Kenya National Tuberculosis Prevalence Survey. PLOS Glob Public Health. 2022;2: e0001272. doi:10.1371/journal.pgph.0001272

6. World Health Organization. WHO Consolidated Guidelines on Tuberculosis. Module 2: Screening - Systematic screening for tuberculosis disease. Geneva, Switzerland: WHO; 2021 Mar.

7. Ho J, Nguyen PTB, Nguyen TA, Tran KH, Van Nguyen S, Nguyen NV, et al. Reassessment of the positive predictive value and specificity of Xpert MTB/RIF: a diagnostic accuracy study in the context of community-wide screening for tuberculosis. Lancet Infect Dis. 2016;16: 1045–1051. doi:10.1016/S1473-3099(16)30067-6
