# Supplementary material for: Potential impact, costs, and benefits of population-wide screening interventions for tuberculosis in Viet Nam: A mathematical modelling study
Source: PLOS Glob Public Health. 2025 Sep 10;5(9):e0005050. doi: 10.1371/journal.pgph.0005050 (PMC12422431; doi:10.1371/journal.pgph.0005050)
Supplement: S5 Table — (PDF) [file pgph.0005050.s014.pdf]

Potential impact, costs, and benefits of population-wide screening interventions for tuberculosis in Viet Nam: a mathematical modelling study

Alvaro Schwalb[1,2,3], Katherine C. Horton[1,2], Jon C. Emery[1,2], Martin J. Harker[1,2,4], Lara Goscé[1,2], Lara D. Veeken[5], Frances L. Garden[6,7], Hai Viet Nguyen[8], Thu-Anh Nguyen[9,10,11,12], Khanh Luu Boi[12], Frank Cobelens[13,14], Greg J. Fox[10,11,12], Van Luong Dinh[15,16], Hoa Binh Nguyen[15,16], Guy B. Marks[6,12,17,18], Rein M.G.J. Houben[1,2]

Affiliations:

1. TB Modelling Group, TB Centre, London School of Hygiene and Tropical Medicine, London, United Kingdom; 2. Department of Infectious Disease Epidemiology, London School of Hygiene and Tropical Medicine, London, United Kingdom; 3. Instituto de Medicina Tropical Alexander von Humboldt, Universidad Peruana Cayetano Heredia, Lima, Peru; 4. Global Health Economics Centre, London School of Hygiene and Tropical Medicine, London, United Kingdom; 5. Department of Internal Medicine and Radboud Community for Infectious Diseases, Radboud University Medical Center, Nijmegen, the Netherlands; 6. South West Sydney Clinical Campuses, University of New South Wales, Sydney, Australia; 7. Ingham Institute of Applied Medical Research, Sydney, Australia; 8. Ministry of Health, Hanoi, Viet Nam; 9. The University of Sydney Vietnam Institute, Ho Chi Minh City, Viet Nam; 10. Faculty of Medicine and Health, University of Sydney, Sydney, Australia; 11. The University of Sydney Institute for Infectious Diseases, Sydney, Australia; 12. Woolcock Institute of Medical Research, Sydney, Australia; 13. Department of Global Health, Amsterdam University Medical Centers, University of Amsterdam, Amsterdam, the Netherlands; 14. Amsterdam Institute for Global Health and Development, Amsterdam, the Netherlands; 15. National Lung Hospital, National Tuberculosis Control Programme, Hanoi, Viet Nam; 16. Hanoi Medical University, Hanoi, Viet Nam; 17. School of Clinical Medicine, University of New South Wales, Sydney, Australia; 18. Burnet Institute, Melbourne, Australia.

Corresponding author: A. Schwalb, London School of Hygiene & Tropical Medicine, Keppel Street, London WC1E 7HT, UK (alvaro.schwalb@lshtm.ac.uk)

S5 Table. Costing estimates for population-wide screening algorithms.

Analysis	Algorithm	Cost per individual (US$)	Distribution
Main analysis	NAAT-only	8.0	Gamma distribution, standard deviation 20% of the mean
	CXR+NAAT	1.7	Gamma distribution, standard deviation 20% of the mean
	CXR-only	1.2	Gamma distribution, standard deviation 20% of the mean
Sensitivity analysis	NAAT-only (US$1 NAAT)	3.0	Gamma distribution, standard deviation 20% of the mean
	CXR+NAAT (US$1 NAAT)	1.3	Gamma distribution, standard deviation 20% of the mean

Costing estimates per individual for different population-wide algorithms. Estimates represent the average cost per individual screened, based on total costs accrued over six years of community-wide screening interventions, informed by the ACT3 trial and the ongoing ACT5 trial [1,2]. Cost components include the number of screening days per year; human resource costs (e.g., technicians, field workers, laboratory staff, administrative staff, and supervisors); the proportion of the population participating and providing sputum samples; the number undergoing NAAT; consumables; screening site setup; and transportation. For the CXR+NAAT algorithm, costs also account for the proportion of CXR deemed abnormal and requiring confirmatory NAAT testing. Unit costs were independently sampled from a gamma distribution. NAAT: Nucleic acid amplification test; CXR: Chest radiography; US$: United States dollar.

References

1. Marks GB, Nguyen NV, Nguyen PTB, Nguyen T-A, Nguyen HB, Tran KH, et al. Community-wide Screening for Tuberculosis in a High-Prevalence Setting. N Engl J Med. 2019;381: 1347–1357. doi:10.1056/NEJMoa1902129

2. Australian New Zealand Clinical Trials Registry. ACTRN12622000115730. In: ANZCTR [Internet]. [cited 6 Feb 2024]. Available: https://anzctr.org.au/Trial/Registration/TrialReview.aspx?ACTRN=12622000115730
