# Supplementary material for: Potential impact, costs, and benefits of population-wide screening interventions for tuberculosis in Viet Nam: A mathematical modelling study
Source: PLOS Glob Public Health. 2025 Sep 10;5(9):e0005050. doi: 10.1371/journal.pgph.0005050 (PMC12422431; doi:10.1371/journal.pgph.0005050)
Supplement: S6 Table — (PDF) [file pgph.0005050.s015.pdf]

**Potential impact, costs, and benefits of population-wide screening interventions for tuberculosis in Viet Nam: a mathematical modelling study**

Alvaro Schwalb[1,2,3], Katherine C. Horton[1,2], Jon C. Emery[1,2], Martin J. Harker[1,2,4], Lara Goscé[1,2], Lara D. Veeken[5], Frances L. Garden[6,7], Hai Viet Nguyen[8], Thu-Anh Nguyen[9,10,11,12], Khanh Luu Boi[12], Frank Cobelens[13,14], Greg J. Fox[10,11,12], Van Luong Dinh[15,16], Hoa Binh Nguyen[15,16], Guy B. Marks[6,12,17,18], Rein M.G.J. Houben[1,2]

**Affiliations:**

1. TB Modelling Group, TB Centre, London School of Hygiene and Tropical Medicine, London, United Kingdom; 2. Department of Infectious Disease Epidemiology, London School of Hygiene and Tropical Medicine, London, United Kingdom; 3. Instituto de Medicina Tropical Alexander von Humboldt, Universidad Peruana Cayetano Heredia, Lima, Peru; 4. Global Health Economics Centre, London School of Hygiene and Tropical Medicine, London, United Kingdom; 5. Department of Internal Medicine and Radboud Community for Infectious Diseases, Radboud University Medical Center, Nijmegen, the Netherlands; 6. South West Sydney Clinical Campuses, University of New South Wales, Sydney, Australia; 7. Ingham Institute of Applied Medical Research, Sydney, Australia; 8. Ministry of Health, Hanoi, Viet Nam; 9. The University of Sydney Vietnam Institute, Ho Chi Minh City, Viet Nam; 10. Faculty of Medicine and Health, University of Sydney, Sydney, Australia; 11. The University of Sydney Institute for Infectious Diseases, Sydney, Australia; 12. Woolcock Institute of Medical Research, Sydney, Australia; 13. Department of Global Health, Amsterdam University Medical Centers, University of Amsterdam, Amsterdam, the Netherlands; 14. Amsterdam Institute for Global Health and Development, Amsterdam, the Netherlands; 15. National Lung Hospital, National Tuberculosis Control Programme, Hanoi, Viet Nam; 16. Hanoi Medical University, Hanoi, Viet Nam; 17. School of Clinical Medicine, University of New South Wales, Sydney, Australia; 18. Burnet Institute, Melbourne, Australia.

**Corresponding author:** A. Schwalb, London School of Hygiene & Tropical Medicine, Keppel Street, London WC1E 7HT, UK (alvaro.schwalb@lshtm.ac.uk)

**S6 Table. Performance of population-wide screening interventions to reach TB prevalence threshold of 100 per 100,000 inhabitants.**

| Screening algorithm | BAU | NAAT | | NAAT+CXR | | CXR |
|---|---|---|---|---|---|---|
| Rounds required to reach threshold | Not reached | 3 annual rounds | | 3 annual rounds | | 2 annual rounds |
| Cumulative TB incidence | 2.25m (95%UI: 1.57-3.04) | 1.39m (95%UI: 0.94-1.89) | | 1.52m (95%UI: 1.04-2.06) | | 0.79m (95%UI: 0.55-1.10) |
| Cumulative TB deaths | 273k (95%UI:123-475) | 160k (95%UI: 70-278) | | 177k (95%UI: 77-308) | | 94k (95%UI:41-163) |
| Cumulative DALYs | 8.12m (95%UI: 5.85-10.83) | 5.13m (95%UI: 3.60-6.82) | | 5.62m (95%UI: 4.04-7.37) | | 3.09m (95%UI: 2.21-4.17) |
| Cumulative TPs diagnosed through screening | N/A | 369k (95%UI: 281-450) | | 313k (95%UI: 232-392) | | 988k (95%UI: 666-1,307) |
| Cumulative FPs diagnosed through screening | N/A | 1,384k (95%UI: 1,033-1,834) | | 617k (95%UI: 360-983) | | 21,107k (95%UI: 16,340-25,722) |
| Unit price of NAAT | N/A | US$8 | US$1 | US$8 | US$1 | N/A |
| Cost of diagnosis/screening | 363m (95%UI: 222-578) | 1,343m (95%UI: 952-1,846) | 641m (95%UI: 471-842) | 639m (95%UI: 478-858) | 548m (95%UI: 398-719) | 311m (95%UI: 230-411) |
| Cost of treatment | 138m (95%UI: 86-209) | 235m (95%UI: 157-344) | | 176m (95%UI: 114-268) | | 1,806m (95%UI: 1,133-2,796) |
| Budget impact | 505m (95%UI: 328-757) | 1,583m (95%UI: 1,183-2,102) | 878m (95%UI: 677-1,113) | 822m (95%UI: 617-1,075) | 722m (95%UI: 548-932) | 2,118m (95%UI: 1,451-3,093) |
| Annual cost of front-loading | N/A | 429m (95%UI: 297-586) | 192m (95%UI: 139-255) | 162m (95%UI: 113-219) | 131m (95%UI: 93-183) | 967m (95%UI: 646-1,466) |
| Annual cost savings | N/A | 8.0m (95%UI: 1.9-15.8) | | 6.9m (95%UI: 0.3-14.8) | | 13.0m (95%UI: 7.1-21.8) |
| ICER compared with BAU (US$ per DALY averted) | N/A | 354 (95%UI: 144-811) | 123 (95%UI: 24-325) | 123 (95%UI: 21-359) | 84 (95%UI: 1-285) | 318 (95%UI: 133-724) |

Epidemiological performance and economic impact of population-wide screening interventions in Viet Nam by algorithm, conducted until the TB prevalence threshold of 100 per 100,000 people is reached. Values represent cumulative outcomes over a 25-year time horizon, extending up to 2050. Budget impact reflects the total cost of screening/diagnosis and treatment for both the intervention and BAU scenarios. The cost of front-loading refers to the average annual screening and treatment cost attributable to the intervention during the implementation period. Annual cost savings are calculated as the average annual difference in BAU-specific diagnosis and treatment costs between the intervention algorithm and the BAU counterfactual. BAU: Business-as-usual; CXR: Chest radiography; DALY: Disability-adjusted life year; FP: False positive; ICER: Incremental cost-effectiveness ratio; NAAT: Nucleic acid amplification test (Xpert MTB/RIF Ultra); TB: Tuberculosis; TP: True positive; UI: Uncertainty interval; US$: United States dollar.
