# Supplementary material for: Potential impact, costs, and benefits of population-wide screening interventions for tuberculosis in Viet Nam: A mathematical modelling study
Source: PLOS Glob Public Health. 2025 Sep 10;5(9):e0005050. doi: 10.1371/journal.pgph.0005050 (PMC12422431; doi:10.1371/journal.pgph.0005050)
Supplement: S7 Table — (PDF) [file pgph.0005050.s016.pdf]

Potential impact, costs, and benefits of population-wide screening interventions for tuberculosis in Viet Nam: a mathematical modelling study

Alvaro Schwalb[1,2,3], Katherine C. Horton[1,2], Jon C. Emery[1,2], Martin J. Harker[1,2,4], Lara Goscé[1,2], Lara D. Veeken[5], Frances L. Garden[6,7], Hai Viet Nguyen[8], Thu-Anh Nguyen[9,10,11,12], Khanh Luu Boi[12], Frank Cobelens[13,14], Greg J. Fox[10,11,12], Van Luong Dinh[15,16], Hoa Binh Nguyen[15,16], Guy B. Marks[6,12,17,18], Rein M.G.J. Houben[1,2]

Affiliations:

1. TB Modelling Group, TB Centre, London School of Hygiene and Tropical Medicine, London, United Kingdom; 2. Department of Infectious Disease Epidemiology, London School of Hygiene and Tropical Medicine, London, United Kingdom; 3. Instituto de Medicina Tropical Alexander von Humboldt, Universidad Peruana Cayetano Heredia, Lima, Peru; 4. Global Health Economics Centre, London School of Hygiene and Tropical Medicine, London, United Kingdom; 5. Department of Internal Medicine and Radboud Community for Infectious Diseases, Radboud University Medical Center, Nijmegen, the Netherlands; 6. South West Sydney Clinical Campuses, University of New South Wales, Sydney, Australia; 7. Ingham Institute of Applied Medical Research, Sydney, Australia; 8. Ministry of Health, Hanoi, Viet Nam; 9. The University of Sydney Vietnam Institute, Ho Chi Minh City, Viet Nam; 10. Faculty of Medicine and Health, University of Sydney, Sydney, Australia; 11. The University of Sydney Institute for Infectious Diseases, Sydney, Australia; 12. Woolcock Institute of Medical Research, Sydney, Australia; 13. Department of Global Health, Amsterdam University Medical Centers, University of Amsterdam, Amsterdam, the Netherlands; 14. Amsterdam Institute for Global Health and Development, Amsterdam, the Netherlands; 15. National Lung Hospital, National Tuberculosis Control Programme, Hanoi, Viet Nam; 16. Hanoi Medical University, Hanoi, Viet Nam; 17. School of Clinical Medicine, University of New South Wales, Sydney, Australia; 18. Burnet Institute, Melbourne, Australia.

Corresponding author: A. Schwalb, London School of Hygiene & Tropical Medicine, Keppel Street, London WC1E 7HT, UK (alvaro.schwalb@lshtm.ac.uk)

S7 Table. Performance of population-wide screening interventions to reach TB prevalence threshold of 20 per 100,000 inhabitants.

Screening algorithm	BAU	NAAT		NAAT+CXR		CXR
**Rounds required to reach threshold**	Not reached	11 annual rounds		12 annual rounds		4 annual rounds
**Cumulative TB incidence**	2.25m (95%UI: 1.57-3.04)	0.62m (95%UI: 0.42-0.85)		0.68m (95%UI: 0.47-0.93)		0.36m (95%UI: 0.26-0.49)
**Cumulative TB deaths**	273k (95%UI:123-475)	63k (95%UI: 26-107)		68k (95%UI: 29-113)		43k (95%UI:20-74)
**Cumulative DALYs**	8.12m (95%UI: 5.85-10.83)	2.92m (95%UI: 2.06-3.84)		3.25m (95%UI: 2.35-4.31)		1.77m (95%UI: 1.30-2.35)
**Cumulative TPs diagnosed through screening**	N/A	711k (95%UI: 520-907)		718k (95%UI: 504-946)		1,262k (95%UI: 852-1,676)
**Cumulative FPs diagnosed through screening**	N/A	5,276k (95%UI: 3,917-6,834)		3,022k (95%UI: 1,754-4,667)		42,402k (95%UI: 32,130-51,051)
**Unit price of NAAT**	N/A	US$8	US$1	US$8	US$1	N/A
**Cost of diagnosis/screening**	363m (95%UI: 222-578)	4,259m (95%UI: 2,782-6,223)	1,671m (95%UI: 1,097-2,380)	2,031m (95%UI: 1,375-2,873)	1,555m (95%UI: 1,061-2,243)	420m (95%UI: 293-577)
**Cost of treatment**	138m (95%UI: 86-209)	540m (95%UI: 336-827)		349m (95%UI: 215-562)		3,494m (95%UI: 2,148-5,477)
**Budget impact**	505m (95%UI: 328-757)	4,801m (95%UI: 3,301-6,780)	2,219m (95%UI:1,586-2,973)	2,368m (95%UI: 1,692-3,259)	1,929m (95%UI: 1,387-2,599)	3,913m (95%UI: 2,598-5,873)
**Annual cost of front-loading**	N/A	426m (95%UI: 290-605)	191m (95%UI: 136-259)	187m (95%UI: 131-260)	150m (95%UI: 106-207)	958m (95%UI: 634-1,447)
**Annual cost savings**	N/A	15.5m (95%UI: 9.0-24.9)		15.2m (95%UI: 8.8-24.7)		16.9m (95%UI: 10.6-26.6)
**ICER compared with BAU (US$ per DALY averted)**	N/A	825 (95%UI: 380-1,713)	328 (95%UI: 143-688)	381 (95%UI: 172-818)	291 (95%UI: 122-643)	537 (95%UI: 240-1,207)

Epidemiological performance and economic impact of population-wide screening interventions in Viet Nam by algorithm, conducted until the TB prevalence threshold of 20 per 100,000 people is reached. Values represent cumulative outcomes over a 25-year time horizon, extending up to 2050. Budget impact reflects the total cost of screening/diagnosis and treatment for both the intervention and BAU scenarios. The cost of front-loading refers to the average annual screening and treatment cost attributable to the intervention during the implementation period. Annual cost savings are calculated as the average annual difference in BAU-specific diagnosis and treatment costs between the intervention algorithm and the BAU counterfactual. BAU: Business-as-usual; CXR: Chest radiography; DALY: Disability-adjusted life year; FP: False positive; ICER: Incremental cost-effectiveness ratio; NAAT: Nucleic acid amplification test (Xpert MTB/RIF Ultra); TB: Tuberculosis; TP: True positive; UI: Uncertainty interval; US$: United States dollar.
