# Supplementary material for: Potential impact, costs, and benefits of population-wide screening interventions for tuberculosis in Viet Nam: A mathematical modelling study
Source: PLOS Glob Public Health. 2025 Sep 10;5(9):e0005050. doi: 10.1371/journal.pgph.0005050 (PMC12422431; doi:10.1371/journal.pgph.0005050)
Supplement: S8 Table — (PDF) [file pgph.0005050.s017.pdf]

Potential impact, costs, and benefits of population-wide screening interventions for tuberculosis in Viet Nam: a mathematical modelling study

Alvaro Schwalb[1,2,3], Katherine C. Horton[1,2], Jon C. Emery[1,2], Martin J. Harker[1,2,4], Lara Goscé[1,2], Lara D. Veeken[5], Frances L. Garden[6,7], Hai Viet Nguyen[8], Thu-Anh Nguyen[9,10,11,12], Khanh Luu Boi[12], Frank Cobelens[13,14], Greg J. Fox[10,11,12], Van Luong Dinh[15,16], Hoa Binh Nguyen[15,16], Guy B. Marks[6,12,17,18], Rein M.G.J. Houben[1,2]

Affiliations:

1. TB Modelling Group, TB Centre, London School of Hygiene and Tropical Medicine, London, United Kingdom; 2. Department of Infectious Disease Epidemiology, London School of Hygiene and Tropical Medicine, London, United Kingdom; 3. Instituto de Medicina Tropical Alexander von Humboldt, Universidad Peruana Cayetano Heredia, Lima, Peru; 4. Global Health Economics Centre, London School of Hygiene and Tropical Medicine, London, United Kingdom; 5. Department of Internal Medicine and Radboud Community for Infectious Diseases, Radboud University Medical Center, Nijmegen, the Netherlands; 6. South West Sydney Clinical Campuses, University of New South Wales, Sydney, Australia; 7. Ingham Institute of Applied Medical Research, Sydney, Australia; 8. Ministry of Health, Hanoi, Viet Nam; 9. The University of Sydney Vietnam Institute, Ho Chi Minh City, Viet Nam; 10. Faculty of Medicine and Health, University of Sydney, Sydney, Australia; 11. The University of Sydney Institute for Infectious Diseases, Sydney, Australia; 12. Woolcock Institute of Medical Research, Sydney, Australia; 13. Department of Global Health, Amsterdam University Medical Centers, University of Amsterdam, Amsterdam, the Netherlands; 14. Amsterdam Institute for Global Health and Development, Amsterdam, the Netherlands; 15. National Lung Hospital, National Tuberculosis Control Programme, Hanoi, Viet Nam; 16. Hanoi Medical University, Hanoi, Viet Nam; 17. School of Clinical Medicine, University of New South Wales, Sydney, Australia; 18. Burnet Institute, Melbourne, Australia.

Corresponding author: A. Schwalb, London School of Hygiene & Tropical Medicine, Keppel Street, London WC1E 7HT, UK (alvaro.schwalb@lshtm.ac.uk)

S8 Table. Performance of population-wide screening interventions with further investigation post-screening.

Screening algorithm	BAU	NAAT	CXR+NAAT	CXR
**Rounds required to reach threshold**	Not reached	6 annual rounds	8 annual rounds	3 annual rounds
**Cumulative TB incidence**	2.25m (95%UI: 1.57-3.04)	1.03m (95%UI: 0.68-1.41)	1.04m (95%UI: 0.69-1.43)	1.22m (95%UI: 0.83-1.67)
**Cumulative TB deaths**	273k (95%UI:123-475)	113k (95%UI: 47-194)	112k (95%UI: 46-196)	140k (95%UI: 62-248)
**Cumulative DALYs**	8.12m (95%UI: 5.85-10.83)	3.99m (95%UI: 2.77-5.31)	4.17m (95%UI: 2.89-5.56)	4.52m (95%UI: 3.22-6.06)
**Cumulative TPs diagnosed through screening**	N/A	490k (95%UI: 377-594)	489k (95%UI: 364-600)	514k (95%UI: 374-670)
**Cumulative FPs diagnosed through screening**	N/A	272k (95%UI: 182-410)	224k (95%UI: 127-383)	3,057k (95%UI: 2,236-4,051)
**Unit price of NAAT**	N/A	8USD	8USD	N/A
**Cost of diagnosis/screening**	363m (95%UI: 222-578)	2,399m (95%UI: 1,655-3,405)	1,243m (95%UI: 816-1,746)	478m (95%UI: 339-641)
**Cost of treatment**	138m (95%UI: 86-209)	137m (95%UI: 91-204)	134m (95%UI: 84-203)	369m (95%UI: 237-553)
**Budget impact**	505m (95%UI: 328-757)	2,540m (95%UI: 1,794-3,567)	1,377m (95%UI: 955-1,885)	921m (95%UI: 688-1,177)
**Annual cost of front-loading**	N/A	387m (95%UI: 262-555)	145m (95%UI: 95-209)	193m (95%UI: 139-262)
**Annual cost savings**	N/A	11.7m (95%UI: 5.8-20.3)	11.6m (95%UI: 5.9-20.2)	9.5m (95%UI: 3.8-18.0)
**ICER compared with BAU (US$ per DALY averted)**	N/A	489 (95%UI: 207-1,105)	219 (95%UI: 73-521)	113 (95%UI: 28-278)

Epidemiological performance and economic impact of population-wide screening interventions with further investigation post-screening in Viet Nam by algorithm, conducted until the TB prevalence threshold of 50 per 100,000 people is reached. Further investigation was applied and costed exclusively for individuals who screened positive under their respective algorithm. Values represent cumulative outcomes over a 25-year time horizon, extending up to 2050. Budget impact reflects the total cost of screening/diagnosis and treatment for both the intervention and BAU scenarios. The cost of front-loading refers to the average annual screening and treatment cost attributable to the intervention during the implementation period. Annual cost savings are calculated as the average annual difference in BAU-specific diagnosis and treatment costs between the intervention algorithm and the BAU counterfactual. BAU: Business-as-usual; CXR: Chest radiography; DALY: Disability-adjusted life year; FP: False positive; ICER: Incremental cost-effectiveness ratio; NAAT: Nucleic acid amplification test (Xpert MTB/RIF Ultra); TB: Tuberculosis; TP: True positive; UI: Uncertainty interval; US$: United States dollar.
