# Supplementary material for: Potential impact, costs, and benefits of population-wide screening interventions for tuberculosis in Viet Nam: A mathematical modelling study
Source: PLOS Glob Public Health. 2025 Sep 10;5(9):e0005050. doi: 10.1371/journal.pgph.0005050 (PMC12422431; doi:10.1371/journal.pgph.0005050)
Supplement: S9 Table — (PDF) [file pgph.0005050.s018.pdf]

Potential impact, costs, and benefits of population-wide screening interventions for tuberculosis in Viet Nam: a mathematical modelling study

Alvaro Schwalb[1,2,3], Katherine C. Horton[1,2], Jon C. Emery[1,2], Martin J. Harker[1,2,4], Lara Goscé[1,2], Lara D. Veeken[5], Frances L. Garden[6,7], Hai Viet Nguyen[8], Thu-Anh Nguyen[9,10,11,12], Khanh Luu Boi[12], Frank Cobelens[13,14], Greg J. Fox[10,11,12], Van Luong Dinh[15,16], Hoa Binh Nguyen[15,16], Guy B. Marks[6,12,17,18], Rein M.G.J. Houben[1,2]

Affiliations:

1. TB Modelling Group, TB Centre, London School of Hygiene and Tropical Medicine, London, United Kingdom; 2. Department of Infectious Disease Epidemiology, London School of Hygiene and Tropical Medicine, London, United Kingdom; 3. Instituto de Medicina Tropical Alexander von Humboldt, Universidad Peruana Cayetano Heredia, Lima, Peru; 4. Global Health Economics Centre, London School of Hygiene and Tropical Medicine, London, United Kingdom; 5. Department of Internal Medicine and Radboud Community for Infectious Diseases, Radboud University Medical Center, Nijmegen, the Netherlands; 6. South West Sydney Clinical Campuses, University of New South Wales, Sydney, Australia; 7. Ingham Institute of Applied Medical Research, Sydney, Australia; 8. Ministry of Health, Hanoi, Viet Nam; 9. The University of Sydney Vietnam Institute, Ho Chi Minh City, Viet Nam; 10. Faculty of Medicine and Health, University of Sydney, Sydney, Australia; 11. The University of Sydney Institute for Infectious Diseases, Sydney, Australia; 12. Woolcock Institute of Medical Research, Sydney, Australia; 13. Department of Global Health, Amsterdam University Medical Centers, University of Amsterdam, Amsterdam, the Netherlands; 14. Amsterdam Institute for Global Health and Development, Amsterdam, the Netherlands; 15. National Lung Hospital, National Tuberculosis Control Programme, Hanoi, Viet Nam; 16. Hanoi Medical University, Hanoi, Viet Nam; 17. School of Clinical Medicine, University of New South Wales, Sydney, Australia; 18. Burnet Institute, Melbourne, Australia.

Corresponding author: A. Schwalb, London School of Hygiene & Tropical Medicine, Keppel Street, London WC1E 7HT, UK (alvaro.schwalb@lshtm.ac.uk)

S9 Table. Performance of population-wide screening interventions with revised CXR sensitivity.

Screening algorithm	BAU	NAAT	CXR+NAAT	CXR
**Rounds required to reach threshold**	Not reached	6 annual rounds	9 annual rounds	3 annual rounds
**Cumulative TB incidence**	2.25m (95%UI: 1.57-3.04)	0.95m (95%UI: 0.63-1.31)	1.00m (95%UI: 0.67-1.35)	0.68m (95%UI: 0.47-0.93)
**Cumulative TB deaths**	273k (95%UI:123-475)	104k (95%UI: 44-184)	105k (95%UI: 45-185)	79k (95%UI: 34-139)
**Cumulative DALYs**	8.12m (95%UI: 5.85-10.83)	3.74m (95%UI: 2.64-4.99)	4.14m (95%UI: 2.91-5.52)	2.79m (95%UI: 2.00-3.74)
**Cumulative TPs diagnosed through screening**	N/A	555k (95%UI: 411-688)	544k (95%UI: 395-698)	985k (95%UI: 672-1,305)
**Cumulative FPs diagnosed through screening**	N/A	2,779k (95%UI: 2,059-3,696)	1,924k (95%UI: 1,122-3,013)	31,354k (95%UI: 24,199-38,227)
**Unit price of NAAT**	N/A	US$8	US$8	N/A
**Cost of diagnosis/screening**	363m (95%UI: 222-578)	2,428m (95%UI: 1,675-3,465)	1,350m (95%UI: 945-1,881)	374m (95%UI: 273-509)
**Cost of treatment**	138m (95%UI: 86-209)	336m (95%UI: 220-511)	272m (95%UI: 158-425)	2,570m (95%UI: 1,623-4,103)
**Budget impact**	505m (95%UI: 328-757)	2,766m (95%UI: 1,965-3,782)	1,639m (95%UI: 1,191-2,196)	2,967m (95%UI: 2,005-4,487)
**Annual cost of front-loading**	N/A	427m (95%UI: 299-599)	159m (95%UI: 110-219)	938m (95%UI: 623-1,448)
**Annual cost savings**	N/A	12.3m (95%UI: 6.5-21.4)	12.2m (95%UI: 5.9-21.0)	14.3m (95%UI: 8.1-23.2)
**ICER compared with BAU (US$ per DALY averted)**	N/A	516 (95%UI: 233-1,073)	283 (95%UI: 114-640)	456 (95%UI: 204-1,062)

Epidemiological performance and economic impact of population-wide screening interventions with revised CXR sensitivity for non-infectious and asymptomatic TB in Viet Nam by algorithm, conducted until the TB prevalence threshold of 50 per 100,000 people is reached. Values represent cumulative outcomes over a 25-year time horizon, extending up to 2050. Budget impact reflects the total cost of screening/diagnosis and treatment for both the intervention and BAU scenarios. The cost of front-loading refers to the average annual screening and treatment cost attributable to the intervention during the implementation period. Annual cost savings are calculated as the average annual difference in BAU-specific diagnosis and treatment costs between the intervention algorithm and the BAU counterfactual. BAU: Business-as-usual; CXR: Chest radiography; DALY: Disability-adjusted life year; FP: False positive; ICER: Incremental cost-effectiveness ratio; NAAT: Nucleic acid amplification test (Xpert MTB/RIF Ultra); TB: Tuberculosis; TP: True positive; UI: Uncertainty interval; US$: United States dollar.
