# Supplementary material for: Potential impact, costs, and benefits of population-wide screening interventions for tuberculosis in Viet Nam: A mathematical modelling study
Source: PLOS Glob Public Health. 2025 Sep 10;5(9):e0005050. doi: 10.1371/journal.pgph.0005050 (PMC12422431; doi:10.1371/journal.pgph.0005050)
Supplement: S10 Table — (PDF) [file pgph.0005050.s019.pdf]

Potential impact, costs, and benefits of population-wide screening interventions for tuberculosis in Viet Nam: a mathematical modelling study

Alvaro Schwalb[1,2,3], Katherine C. Horton[1,2], Jon C. Emery[1,2], Martin J. Harker[1,2,4], Lara Goscé[1,2], Lara D. Veeken[5], Frances L. Garden[6,7], Hai Viet Nguyen[8], Thu-Anh Nguyen[9,10,11,12], Khanh Luu Boi[12], Frank Cobelens[13,14], Greg J. Fox[10,11,12], Van Luong Dinh[15,16], Hoa Binh Nguyen[15,16], Guy B. Marks[6,12,17,18], Rein M.G.J. Houben[1,2]

Affiliations:

1. TB Modelling Group, TB Centre, London School of Hygiene and Tropical Medicine, London, United Kingdom; 2. Department of Infectious Disease Epidemiology, London School of Hygiene and Tropical Medicine, London, United Kingdom; 3. Instituto de Medicina Tropical Alexander von Humboldt, Universidad Peruana Cayetano Heredia, Lima, Peru; 4. Global Health Economics Centre, London School of Hygiene and Tropical Medicine, London, United Kingdom; 5. Department of Internal Medicine and Radboud Community for Infectious Diseases, Radboud University Medical Center, Nijmegen, the Netherlands; 6. South West Sydney Clinical Campuses, University of New South Wales, Sydney, Australia; 7. Ingham Institute of Applied Medical Research, Sydney, Australia; 8. Ministry of Health, Hanoi, Viet Nam; 9. The University of Sydney Vietnam Institute, Ho Chi Minh City, Viet Nam; 10. Faculty of Medicine and Health, University of Sydney, Sydney, Australia; 11. The University of Sydney Institute for Infectious Diseases, Sydney, Australia; 12. Woolcock Institute of Medical Research, Sydney, Australia; 13. Department of Global Health, Amsterdam University Medical Centers, University of Amsterdam, Amsterdam, the Netherlands; 14. Amsterdam Institute for Global Health and Development, Amsterdam, the Netherlands; 15. National Lung Hospital, National Tuberculosis Control Programme, Hanoi, Viet Nam; 16. Hanoi Medical University, Hanoi, Viet Nam; 17. School of Clinical Medicine, University of New South Wales, Sydney, Australia; 18. Burnet Institute, Melbourne, Australia.

Corresponding author: A. Schwalb, London School of Hygiene & Tropical Medicine, Keppel Street, London WC1E 7HT, UK (alvaro.schwalb@lshtm.ac.uk)

S10 Table. Cost-effectiveness of population-wide screening interventions for TB.

Analysis type	Screening algorithm	DALYs averted compared to BAU	Additional costs (US$) compared to BAU	Incremental DALYs	Incremental costs (US$)	ICER (US$ per DALY averted)
Reducing the unit price of NAAT cartridges to US$1	CXR+NAAT	4.28m (95%UI: 2.93-6.16)	0.71b (95%UI: 0.35-1.11)	4.28m (95%UI: 2.93-6.16)	0.71b (95%UI: 0.35-1.11)	167 (95%UI: 57-380)
	NAAT	4.38m (95%UI: 2.97-6.19)	0.83b (95%UI: 0.44-1.27)	Removed due to extended dominance with respect to CXR-only		
	CXR	5.94m (95%UI: 4.18-7.97)	2.44b (95%UI: 1.41-3.88)	1.52m (95%UI: 0.79-2.37)	1.61b (95%UI: 0.51-3.06)	1,057 (95%UI: 642-1,291)
TB prevalence threshold of 100 per 100,000 people	CXR+NAAT	2.55m (95%UI: 1.53-3.92)	0.31b (95%UI: 0.08-0.55)	2.55m (95%UI: 1.53-3.92)	0.31b (95%UI: 0.08-0.55)	123 (95%UI: 21-359)
	NAAT	3.02m (95%UI: 1.93-4.47)	1.07b (95%UI: 0.64-1.57)	Removed due to extended dominance with respect to CXR-only		
	CXR	5.06m (95%UI: 3.56-6.86)	1.61b (95%UI: 0.91-2.58)	2.49m (95%UI: 1.57-3.54)	1.31b (95%UI: 0.59-2.25)	528 (95%UI: 376-636)
TB prevalence threshold of 20 per 100,000 people	CXR+NAAT	4.89m (95%UI: 3.34-6.92)	1.86b (95%UI: 1.19-2.73)	4.89m (95%UI: 3.34-6.92)	1.86b (95%UI: 1.19-2.73)	381 (95%UI: 172-818)
	NAAT	5.19m (95%UI: 3.65-7.35)	4.29b (95%UI: 2.79-6.26)	Removed due to simple dominance with respect to CXR-only		
	CXR	6.32m (95%UI: 4.49-8.66)	3.39b (95%UI: 2.08-5.43)	1.46m (95%UI: 0.81-2.31)	1.52b (95%UI: 0.12-3.62)	1,036 (95%UI: 148-1,567)
Performance of using Xpert MTB/RIF in a NAAT-only algorithm	NAAT (Xpert MTB/RIF)	4.29m (95%UI: 2.93-6.16)	2.96b (95%UI: 1.89-4.41)	Removed due to simple dominance with respect to CXR+NAAT		
	CXR+NAAT	4.29m (95%UI: 2.86-6.14)	0.97b (95%UI: 0.52-1.49)	4.29m (95%UI: 2.86-6.14)	0.97b (95%UI: 0.52-1.49)	225 (95%UI: 85-520)
	NAAT (Xpert MTB/RIF Ultra)	4.36m (95%UI: 3.09-6.23)	2.25b (95%UI: 1.45-3.31)	Removed due to extended dominance with respect to CXR-only		
	CXR	5.94m (95%UI: 4.18-7.97)	2.44b (95%UI: 1.41-3.88)	1.61m (95%UI: 0.86-2.56)	1.49b (95%UI: 0.34-2.87)	927 (95%UI: 393-1,124)

Performance of further investigation post-screening	CXR	3.59m (95%UI: 2.43-5.23)	0.41b (95%UI: 0.15-0.68)	3.59m (95%UI: 2.43-5.23)	0.41b (95%UI: 0.15-0.68)	113 (95%UI: 28-278)
	CXR+NAAT	3.96m (95%UI: 2.61-5.83)	0.87b (95%UI: 0.43-1.36)	0.37m (95%UI: 0.00-1.15)	0.46b (95%UI: 0.03-1.00)	1,293 (95%UI: 1,153-1,555)
	NAAT	4.14m (95%UI: 2.77-5.99)	2.03b (95%UI: 1.24-3.06)	0.16m (95%UI: 0.00-0.48)	1.16b (95%UI: 0.23-2.23)	6,183 (95%UI: 5,165-10,441)
Revised CXR sensitivity for non-infectious and asymptomatic TB	CXR+NAAT	3.98m (95%UI: 2.64-5.73)	1.12b (95%UI: 0.65-1.69)	3.98m (95%UI: 2.64-5.73)	1.12b (95%UI: 0.65-1.69)	283 (95%UI: 113-640)
	NAAT	4.36m (95%UI: 3.09-6.23)	2.25b (95%UI: 1.45-3.31)	Removed due to extended dominance with respect to CXR-only		
	CXR	5.35m (95%UI: 3.77-7.33)	2.44b (95%UI: 1.49-4.00)	1.34m (95%UI: 0.62-2.16)	1.32b (95%UI: 0.22-2.95)	962 (95%UI: 750-1,447)

Cost-effectiveness sensitivity analyses of population-wide screening interventions in Viet Nam by algorithm, conducted until the TB prevalence threshold of 50 per 100,000 people is reached unless stated otherwise. Values represent cumulative outcomes over a 25-year time horizon, extending up to 2050. BAU: Business-as-usual; CXR: Chest radiography with computer-aided detection software interpretation; DALY: Disability-adjusted life year; ICER: Incremental cost-effectiveness ratio; NAAT: Nucleic acid amplification test (primarily Xpert MTB/RIF Ultra unless specified otherwise); TB: Tuberculosis; UI: Uncertainty interval; US$: United States dollar.
