# Supplementary material for: Potential impact, costs, and benefits of population-wide screening interventions for tuberculosis in Viet Nam: A mathematical modelling study
Source: PLOS Glob Public Health. 2025 Sep 10;5(9):e0005050. doi: 10.1371/journal.pgph.0005050 (PMC12422431; doi:10.1371/journal.pgph.0005050)
Supplement: Attachment — Submitted filename: Changes to preprint.docx [file pgph.0005050.s020.docx]

**Changes to preprint**

The submitted manuscript has been slightly revised as compared to the version of the manuscript available as a preprint (<https://www.medrxiv.org/content/10.1101/2024.12.30.24319770v1>). Below, there is a point-by-point account of the changes:

1. Reference formatting has been changed to match *PLoS Global Public Health* requirements.
2. Added “Hanoi Medical University, Hanoi, Viet Nam” affiliation to co-authors Dinh Van Luong and Hoa Binh Nguyen.
3. Added sentence to reference T. Parran on advocating for investing in TB: “*Advocating for TB eradication on economic grounds is compelling [38], as trends suggesting that cost savings will persist alongside the long-lasting societal benefits of the intervention beyond the time horizon.*” (Line 354-356).
4. Edited typo in Figure 1 legend to describe the model: “*The model is depicted using nine compartments across the spectrum of TB.*” (Line 678-679)
5. Added Figure 3 in manuscript.
6. Added line numbering throughout.

Please let us know if we can provide any additional information. We look forward to hearing from you at your earliest convenience.

Warm wishes,

Alvaro Schwalb, for the authors
