# Supplementary material for: Potential impact, costs, and benefits of population-wide screening interventions for tuberculosis in Viet Nam: A mathematical modelling study
Source: PLOS Glob Public Health. 2025 Sep 10;5(9):e0005050. doi: 10.1371/journal.pgph.0005050 (PMC12422431; doi:10.1371/journal.pgph.0005050)
Supplement: Attachment — Submitted filename: Response to Reviewers.docx [file pgph.0005050.s021.docx]

**Response to reviewer comments**

We thank the journal and the reviewer for their constructive review of our work. We are also grateful for the extension provided, which allowed us the time to carefully consider and address the comments. We have revised the manuscript accordingly. Please find the responses below:

**Reviewer #1:**

1. **The description of screening eligibility criteria (p. 5, lines 143–145) could be slightly more explicit regarding whether individuals previously treated for TB are included in screening rounds.**

We thank the reviewer for this comment. In our model, we assumed that the entire adult population, including individuals previously treated for TB, would be eligible for population-wide screening. As such, the screening algorithms incorporate a specific test positivity for this group (i.e., the *Treated* state). However, individuals undergoing TB treatment were assumed to be ineligible for screening during intervention years. We have amended the manuscript to clarify this point: “*The population was uniformly screened across all model states (except for those undergoing TB treatment), with no increased probability of screening for individuals with TB disease.*” [Methods, lines 146–148].

2. **It would be useful to specify whether the assumption that all infectious TB cases can provide sputum samples (p. 6, lines 147–148) is based on empirical data.**

We thank the reviewer for this helpful suggestion. This assumption is supported by empirical data from a community-based study in Kampala, Uganda, where nearly 100% of screened individuals were able to produce analysable samples for testing using Xpert MTB/RIF Ultra (Kendall et al. *Clin Infect Dis* 2021). We also note that the 60% figure is based on ACT3 data. The changes have been incorporated in the manuscript: “*Additionally, we assumed that every individual with infectious TB (i.e. asymptomatic and symptomatic) would be able to provide an analysable sample [34], while only 60% of those with other model states would be able to do so [16].*” [Methods, lines 148–150].

3. **The results section highlights a high false-positive rate in the CXR-only strategy (TP:FP ratio of 1:27, p. 9, lines 283–284). While the discussion acknowledges the ethical and financial implications, it may be beneficial to provide a brief estimate of potential adverse treatment effects (e.g., hepatotoxicity risks).**

Thank you for the comment. We fully acknowledge the clinical, ethical, and financial implications of overtreatment, particularly the risk of adverse events such as hepatotoxicity. While our modelling framework does not explicitly capture the occurrence of adverse treatment effects, we have addressed this limitation in the discussion, where we cite relevant literature highlighting the potential harms, and how could this potentially offset the DALYs averted. As the CXR-only strategy was evaluated primarily as a theoretical scenario to explore the achievable impact, we chose not to model treatment-related morbidity in detail. We highlight this issue in the Discussion [lines 368-371].

4. **The discussion could further explore how confirmatory testing could be integrated to reduce overtreatment without undermining screening effectiveness.**

We thank the reviewer for this valuable comment. In our fourth sensitivity analysis, we explored the impact of further assessment following screening as a proxy for confirmatory testing. This scenario significantly reduced overtreatment, particularly in the CXR-only algorithm, while retaining much of the screening effectiveness. We have now made this analysis more explicit in the manuscript: “*In our analysis, this approach reduced FPs while maintaining much of the epidemiological benefit.*” [Discussion, lines 409–410].

5. **The study focuses on Vietnam, but given the global burden of TB, a short discussion on the generalizability of these findings to other high-burden settings (e.g., sub-Saharan Africa, India) would enhance the study’s impact.**

We thank the reviewer for this excellent suggestion. While our model was calibrated to Viet Nam, the findings may be relevant to other high TB burden settings. We have expanded accordingly: “*Although our model was calibrated to Viet Nam TB epidemiology, the underlying framework and conclusions, particularly regarding the trade-offs between screening performance, intensity, and cost-effectiveness, are relevant to other high-burden settings. However, applying these findings elsewhere may require additional consideration of context-specific structural risk factors such as HIV.*” [Discussion, lines 426-430].

6. **Some figures and tables could benefit from clearer legends, particularly Table 1, where test performance parameters could be more explicitly described.**

Thank you for this comment. We reviewed all figures and tables with a focus on improving clarity, with minor clarifications made where appropriate.

7. **A few minor grammatical inconsistencies (e.g., "underscoring the importance of introducing proactive measures" could be "highlighting the need for proactive measures") should be addressed.**

Thank you for this comment. We have reviewed the manuscript again for grammatical inconsistencies.

**Reviewer #2:**

8. **My biggest concern is that this analysis overstates confidence of diagnostic accuracy estimates (particularly for the CXR-only screening) and makes several inaccurate assumptions about accuracy across different states (including for the algorithms that include NAAT). Specifically:**
   a. **CXR positivity estimates are largely ungrounded in data. These will depend heavily on how TB is identified by CXR/CAD, yet the current value is just a midpoint between other estimates and is assigned an overly narrow uncertainty range. I would like to see some consideration of what CAD thresholds are being considered (with 85% sensitivity for symptomatic TB and 91.5% specificity for all TB), and what proportion of the “unconfirmed” state those cutoffs are really likely to identify. I would also like to see more careful sensitivity analysis around this (with wider uncertainty).**

We thank the reviewer for raising these points. We acknowledge that CAD thresholds can significantly influence both sensitivity and specificity, but these are highly variable between settings. We also recognise that the CXR test positivity values for the non-infectious and asymptomatic TB states are not grounded in direct empirical data, although estimates for all other model states were data-informed. This limitation motivated the inclusion of the fifth sensitivity analysis in our study, which explored more conservative, data-driven assumptions for CXR positivity in the non-infectious and asymptomatic states. This analysis showed limited impact on the key epidemiological and economic outcomes. We have now clarified in the manuscript that the assumed test positivity values do not correspond to a particular CAD threshold: “*CXR positivity values do not correspond to a specific CAD threshold and are explored further in the sensitivity analyses.*” [Table 1 legend, lines 671–672].

b. **NAAT positivity among the susceptible/infected/cleared is likely overstated. The estimate of specificity is from a population that included individuals with unconfirmed TB and recovered/treated TB among the TB-negatives – and it is likely that these groups accounted for most of the observed “false positive” Xpert Ultra results.**

Thank you for this comment. We agree that Xpert Ultra positivity among individuals in the susceptible, infected, or cleared states is likely overestimated, potentially inflating false-positive rates due to trace results. However, this reinforces our results as conservative, and notably, the intervention remains cost-effective even under these conditions. Given current limitations in the available data, we opted not to reparameterise the model but acknowledge that more refined empirical data would strengthen future modelling efforts. We also explored this issue in the fourth sensitivity analysis where we consider a final evaluation with high specificity in the non-disease states.

c. **Sensitivity of NAAT for symptomatic TB is based on data from a care-seeking population, and the authors should instead use data from community screening studies or prevalence surveys to estimate the (lower) sensitivity among all symptom-screen-positive TB.**

We thank the reviewer for this important observation. We acknowledge that NAAT sensitivity for symptomatic TB is likely lower in community screening settings compared to care-seeking populations. However, there are currently no available data on Xpert Ultra sensitivity in community-based use that allow for reliable parameterisation. For instance, the study in Kampala, Uganda (Kendall et al., *Clin Infect Dis* 2021) reports that “*the number of Ultra-negative individuals who underwent culture was too small to estimate sensitivity with precision*”. Given the absence of such data, we used estimates from clinical settings, recognising that these likely overestimates true sensitivity in community-based contexts. We have now made this limitation more explicit in the manuscript and acknowledge that the assumed sensitivity is optimistic: “*Similarly, relying on care-seeking, clinic-based populations to inform NAAT sensitivity for symptomatic TB likely overestimates its performance in community-based screening and reflects an optimistic scenario.*” [Discussion, lines 437–440]. Of note, while a lower sensitivity would reduce the yield of NAAT-based screening and increase the budget impact, the ICER may not change substantially. Additional rounds of screening required to reach prevalence thresholds would still identify individuals with earlier states of TB, thereby contributing to improved health outcomes.

d. **CXR positivity is assumed to be higher for unconfirmed TB than for treated TB, but the inverse could be true (that people who have progressed to symptomatic sputum+ TB and been treated may have persistent lung pathology and higher CAD scores than those with early sputum-negative TB.**
   Thank you for this observation. We acknowledge that individuals with treated TB may exhibit persistent radiographic abnormalities, such as fibrotic scars or calcifications, which could potentially influence CAD scores. However, other lesions, such as consolidation, cavitation, and nodular opacities, are more indicative of active disease (Bhalla et al. *Indian J Radiol Imaging* 2015) and are more likely to be flagged by CAD. In our model, we assumed higher CXR positivity for non-infectious TB (previously “unconfirmed”) compared to treated TB, reflecting the presence of active lesions in the former.
e. **It isn’t clear to me how NAAT prevalence among presumptive TB patients is relevant as a basis for estimating NAAT sensitivity in unconfirmed TB. Relevant data would focus on individuals with positive NAAT and negative culture, ideally from a community screening context, and with either longitudinal follow-up for outcomes off treatment or with other diagnostic testing (e.g. CT/PET) to determine presence of active TB.**

We thank the reviewer for this comment. We agree that, ideally, NAAT sensitivity for non-infectious TB (previously “unconfirmed”) would be informed by longitudinal follow-up data; however, such data remain limited. Following the ICE-TB classification (Coussens et al., *Lancet Respir Med* 2024), our non-infectious TB category includes both asymptomatic and symptomatic presentations. As such, the symptomatic subset overlaps with what is typically considered “presumptive TB,” which justifies the use of data from that setting to inform test positivity assumptions. We also note that NAAT positivity in this context reflects bacteriological evidence. We have clarified these points in the revised supplementary material: “*For Non-infectious TB, the NAAT test positivity was informed by data from presumptive TB populations, given partial overlap in symptomatic individuals.*” [Table S3 legend, lines 241–243]. We have also mentioned that data from community screening would also improve those estimates: “*Direct data from community screening would provide improved estimates of test positivity*.” [Discussion, lines 436-437].

f. **Correlations: The assumption of test independence (i.e. multiplying individual probabilities of positivity for each test) will underestimate sensitivity of the two-step algorithm, because TB with higher sputum bacillary burden is also more likely to have abnormal x-rays. I would like to see the accuracy estimates account for this. This is tricker to represent in a compartmental modelling framework, but tests are also likely to be correlated over time, such that the same people may screen falsely positive or falsely negative on multiple rounds of screening (reducing impact but potential limiting overtreatment). This could be a discussion point.**

Thank you for picking this up. We agree that the assumption of test independence likely underestimates the true sensitivity of two-step screening algorithms, as individuals with higher bacillary burden are more likely to test positive on CXR. While we recognise that incorporating test correlation would be more realistic, doing so within our deterministic compartmental modelling framework would have required additional complexity. We opted for a simplistic approach for the two-step positivity. As suggested, we have now added this point to the discussion: “*In contrast, sensitivity is likely underestimated in the CXR+NAAT algorithm, as we assumed test positivity independence between diagnostic tools, whereas individuals with higher bacillary burden are more likely to test positive on CXR.*” [Discussion, lines 440-442].

g. **The assumptions about sputum production seem implausible: Assuming that every person with infectious TB can produce sputum will overestimate the impact of strategies that include NAAT, while assuming that only 60% of the TB-negative can produce sputum (considerably lower than in e.g. the TREATS study and others that tried to get a NAAT sample from everyone) will underestimate costs.**

Thank you. We address this in a similar comment, see response to item 2.

9. **A major area of uncertainty, which isn’t adequately explored in current sensitivity analyses, is natural history as it relates to the unconfirmed state. For example:**
   a. **How does the impact of the CXR intervention depend on the timing of the infected -> unconfirmed TB transition or the % of infected individuals who pass through an unconfirmed (Xray-positive sputum-negative) state?**

Thank you for this question. In our compartmental model, the transitions between states, including from infected to non-infectious TB (previously “unconfirmed”), occur continuously at fixed rates, and the screening intervention is applied uniformly across all model states over time. As such, the timing of these transitions does not directly influence the impact of the interventions, and all individuals in the non-infectious TB state are eligible for screening at any point. We therefore do not expect this to have affected the results meaningfully.

b. **For the rate of progression from unconfirmed to asymptomatic, the range considered is narrow, and the value isn’t really informed by the calibration process. I imagine that results depend heavily on this value?**

We thank the reviewer for this comment. The rate of progression from non-infectious (previously “unconfirmed”) to asymptomatic TB was informed by the progression rates from Horton et al. (*Proc Natl Acad Sci* 2023). While included in the calibration process, its posterior range did not shift substantially (see Supplementary Material Table S2, pg 12), reflecting the compatibility of our model structure, previous work and the Viet Nam data. While the parameter influences the size of the non-infectious compartment, it is one of multiple progression and regression parameters which all combine to drive model behaviour, together with the test positivity of screening algorithms.

c. **The authors are defining the unconfirmed state in this analysis such that only a proportion have positive x-rays – but their earlier work to estimate related parameters used cohorts defined by x-ray findings. It is unclear how reliably those progression risks can be applied to the definitions they are using here.**

We thank the reviewer for this valid point raised. In earlier work (Sossen et al*. Lancet Respir Med* 2023; Richards et al. *Lancet Glob Health* 2021), estimates of progression risks were indeed informed by cohorts defined by CXR from the pre-chemotherapy era. The ideal data would have been obtained from long-term follow-up using PET/CT evidence, but that was not available at the time of the review. While these data remain rare, in a recent study (Esmail et al. *medRxiv* 2023), PET/CT based-evidence of progression showed broad concordance with the rates previously estimated using CXR-defined cohorts. In this analysis, we aimed to preserve the conceptual definition of the non-infectious TB (previously “unconfirmed”) state as the presence of macroscopic pathology, which may or may not be radiologically detectable, and therefore applied less than 100% CXR positivity. In the context of imperfect data, we believe our approach reflects a pragmatic synthesis of the available evidence.

10. **The diagnostic delineation between different states should be more clearly described. E.g. is the line between unconfirmed and asymptomatic a positive sputum culture?**

We thank the reviewer for this observation. We have clarified that the transition from non-infectious (previously “unconfirmed”) to asymptomatic TB is defined by the presence of bacteriological evidence (not necessarily sputum culture) and associated infectiousness, while the transition from asymptomatic to symptomatic TB is determined by the onset of symptoms. The manuscript has been updated accordingly: “*The transition from non-infectious to asymptomatic TB was defined by the presence of bacteriological evidence of disease, such as a positive sputum smear, nucleic acid amplification test (NAAT), or culture, indicating infectiousness; the transition from asymptomatic to symptomatic TB was determined by the presence of self-reported symptoms.*” [Methods, lines 110–114].

11. **Considering only the median projections seems to underrepresent uncertainty of results. What is the rationale for this, and how much did the full posterior vary?**

Thank you for this comment. We believe this may be a misunderstanding. The median projection was used only to determine whether a given screening strategy reached the TB prevalence threshold (i.e. <100, <50, or <20 per 100,000). In all other results, figures, and tables, we present our estimates with their associated 95% uncertainty intervals. We have now made this more explicit in the text: “*We used the median value of the model outputs and considered the threshold met if it fell below or within 10% above the target. Full uncertainty intervals were presented for all outcomes throughout the results.*” [Methods, lines 162–165].

12. **The secondary analysis modelling “further investigation” after a positive screen is poorly explained. After some thought, I can accept that clinicians might treat everyone with either a prolonged cough or positive confirmatory microbiological testing (e.g. culture), but this could be better explained. It’s also unclear why the cost is $2 per person, and whether this applies to all people who are screened (which seems high) or to only those who screened positive (in which case it is quite low given that the assumed 100% positivity among asymptomatic and symptomatic TB would require culture).**

We thank the reviewer for this helpful comment. We have clarified in the manuscript that this sensitivity analysis was intended to explore the potential role of additional clinical evaluation following a positive screen, rather than to model a specific diagnostic algorithm. We now describe this as “*further evaluation by a clinician based on existing clinical, radiological, or microbiological information*” rather than formal confirmatory testing such as culture [Methods, lines 238-239]. The aim was to reflect a more conservative approach to treatment initiation that could plausibly occur in practice. The assumed cost of US$2 per individual screened positive was selected as a nominal placeholder in the absence of empirical data and is not intended to reflect the cost of any specific diagnostic. We have revised the relevant text to better reflect this intent: “*In absence of clear data from the literature, we used performance of prolonged cough as a proxy (see Table S3) and assumed a fixed cost of US$2 per; this value was illustrative and not intended to reflect the cost of any specific diagnostic test.*” [Methods, lines 242-244].

13. **The authors are trying to align with WHO terminology, but I wouldn’t use “unconfirmed” to describe sputum-negative active disease (previously called “minimal” or “noninfectious” TB disease). An important difference between the WHO framework (intended for programmatic relevance) and other recent classification schemes is that the WHO categories represent what tests *were done* and not what the results of more extensive testing *would have been*. Therefore, a lot of culture-positive TB may be “unconfirmed” in the WHO framework because it is NAAT-negative, or is sputum smear negative (where NAAT is unavailable), or is in a person who had trouble producing sputum and was diagnosed by x-ray. That is different from the state that precedes asymptomatic TB in this model, which is intended to represent sputum (culture) negative TB specifically. To align with WHO terminology but better represent biological states over diagnostic modality, I suggest “asymptomatic bacteriologically negative”, “asymptomatic bacteriologically positive”, and “symptomatic”.**

Thank you for this comment. We agree that the term “unconfirmed” may conflate diagnostic uncertainty with biological state and have now clarified in the Methods that this model state represents bacteriologically negative TB. We have opted to describe its operational definition in the model as non-infectious TB. We have strengthened the definition to make this distinction explicit: “*The three disease states were defined as follows: (i) non-infectious TB, representing individuals with inflammatory pathology but without bacteriological evidence of TB disease, infectiousness, or symptoms.*” [Methods, lines 105–107].

14. **Abstract: it’s odd to say “we designed” screening algorithms. Perhaps “simulated” or “represented”?**

We agree with the reviewer that “simulated” more accurately reflects our methodological approach. We have revised the abstract accordingly: “*We simulated three population-wide screening algorithms from 2025*” [Abstract, lines 34–35].

15. **The “revised CXR sensitivity” assumptions should be explained briefly in the text before presenting the corresponding results; I could find them only in a footnote of a supplemental table.**

Thank you for this observation. We agree that this sensitivity analysis could be more clearly referenced in the main text. While the assumptions were described in the methods, we have now made this more explicit: “*Finally, we explored a revised CXR sensitivity scenario, assuming reduced test positivity for non-infectious and asymptomatic TB, equivalent to its performance in individuals who have recovered or been treated*” [Methods, lines 244–247].

Please let us know if we can provide any additional information. We look forward to hearing from you at your earliest convenience.

Warm wishes,

Alvaro Schwalb, for the authors
