# Supplementary material for: Potential impact, costs, and benefits of population-wide screening interventions for tuberculosis in Viet Nam: A mathematical modelling study
Source: PLOS Glob Public Health. 2025 Sep 10;5(9):e0005050. doi: 10.1371/journal.pgph.0005050 (PMC12422431; doi:10.1371/journal.pgph.0005050)
Supplement: Attachment — Submitted filename: Response to Reviewer.docx [file pgph.0005050.s022.docx]

**Response to reviewer comments**

We thank the journal and the reviewer for their constructive review of our work. We have revised the manuscript accordingly. Please find the responses below:

**Reviewer #1:**

1. **I have carefully reviewed the revised version and find that the authors have addressed previous comments thoroughly. The study is well-conducted, methodologically sound, and provides important insights into TB control strategies in Viet Nam and similar high-burden settings. I recommend the manuscript for acceptance in its current form.**

We thank the reviewer for their time and thoughtful evaluation of our manuscript.

**Reviewer #2:**

2. **In response to comments by both reviewers about sputum production, the authors cite Kampala and ACT3 screening studies, but neither of those studies support the assumption that sputum production differs by TB status or that everyone with TB can produce sputum. These parameter values warrant more careful consideration and revision.**

We thank the reviewer for this comment. We acknowledge that neither the ACT3 nor Kampala studies directly demonstrate that sputum production varies by TB status, nor do they support the assumption that all individuals with TB can produce sputum. In our model, we assumed that sputum production is unevenly distributed by TB status, and we have now made it more explicit in the manuscript that this is an assumption rather than a data-driven input: “*Additionally, we made the simplifying assumption that sputum production is unevenly distributed by TB status: all individuals with infectious TB (i.e. asymptomatic and symptomatic) were assumed to be able to provide an analysable sample, while only 60% of those with other model states would be able to do so.*” [Methods, lines 148-151]. Additionally, we note in the discussion that the unknown distribution of sputum production across TB states highlights the importance of exploring sputum-independent sample collection approaches, such as tongue swabs, in future screening strategies: “*Moreover, the unknown distribution of sputum production across TB states highlights the need for sputum-independent diagnostic approaches, such as tongue swabs.*” [Discussion, lines 448-450].

3. **Authors respond to questions about their CXR accuracy estimates by saying they don’t correspond to any particular CAD cutoff, but they don’t address the more fundamental question of whether *any* cutoff would simultaneously have the combination of sensitivity and specificity values that they chose. As the authors used prevalence surveys as the basis for their CXR specificity estimate, and most prevalence surveys did both CXR and microbiological testing for everyone with symptoms, it should be possible to estimate the sensitivity of CXR (using the same diagnostic cutoff) in those same surveys.**

We thank the reviewer for raising this important point. We have now taken a simple approach to estimate the implied diagnostic accuracy of CXR in our model, based on the point estimates for CXR test positivity assigned to each TB disease state and the distribution of individuals across states. These assumptions correspond to an overall diagnostic performance with an approximate sensitivity of 74% and specificity of 87%. We acknowledge that this does not directly map to any fixed CAD score threshold. For comparison, data from Mungai et al. (*PLoS Glob Public Health* 2022) evaluating CAD4TBv6 show that a score threshold of ~54 achieves around 93% sensitivity and 86% specificity for bacteriologically confirmed TB. Our assumed sensitivity, therefore, reflects a more conservative value than might be expected at typical CAD thresholds. We also note that performance estimates from human CXR reading (Mendelsohn et al. *medRxiv* 2025) vary substantially, and the relationship between CXR sensitivity/specificity and bacteriological confirmation is still evolving. Our modelled assumptions reflect generalised CXR performance across TB states rather than being anchored to a specific tool or operational threshold. We have clarified this in the revised manuscript and acknowledge that this remains a moving field, with further evaluation needed to define the role of CXR: “*Lastly, the sensitivity and specificity values used in our model do not correspond to any particular CAD threshold. However, this remains a rapidly evolving field, and the optimal role of CXR—particularly in prevalence surveys and community-based screening—requires further evaluation.*” [Discussion, lines 416-419].

4. **While it is true that data are sparse for estimating the sensitivity of NAAT in a screening context, the authors should, at a minimum, make clear in Table 1 that they are estimating sensitivity based on care-seeking populations.**

We thank the reviewer for this helpful suggestion. We have now expanded the description in Table 1 to clarify that the sensitivity of NAAT for pulmonary TB is based on care-seeking populations, in line with detail in the supplementary material (S3 Table). The source is described as “*Sensitivity for pulmonary TB from individuals in primary care facilities and local hospitals*”.

5. **Regarding correlations, I would like to see the authors revise their sensitivity and specificity estimates for the CXR + NAAT algorithm to account for expected test non-independence. (The correlations of an individual’s test results over time between different screening rounds are the aspect of this that I think can be a discussion point.)**

We thank the reviewer for the comment. As mentioned in our previous response, we agree that the assumption of test independence likely underestimates the true sensitivity of the CXR+NAAT algorithm, as individuals with higher bacillary burden are more likely to test positive on both tests. However, the test positivity values used for infectious TB are already at the higher end of available estimates, so while we recognise the issue, we feel the results are likely robust to this. This assumption and its potential implications have been acknowledged as a limitation in the manuscript: “*In contrast, assuming independence between test results in the CXR+NAAT algorithm may underestimate sensitivity and thus the effectiveness of the algorithm, as individuals with higher bacillary burden are more likely to test positive on both tests.*” [Discussion, lines 444-446]. Regarding test result correlations over time across multiple screening rounds, we agree that this is an interesting and relevant issue. As prevalence drops through rounds, it is likely that test performance will change as well (e.g. as per Murad et al., CMAJ 2023; Veeken et al., J Infect Dis 2025), as will the correlated performance of a multi-test algorithm. We have added a line to reflect this: “*In addition, the correlation of test results across screening rounds—as TB prevalence declines over time—may influence algorithm performance and merits further investigation.*” [Discussion, lines 446-448].

6. **Authors are inconsistent regarding the symptom status of “non-infectious TB”. They state in the methods that these are “*without bacteriological evidence of TB disease, infectiousness, *or symptoms**”, which I think is the appropriate definition based on their model’s structure and how they estimated natural history parameters. But they contradict this choice when trying to justify their use of presumptive-TB patients to estimate NAAT sensitivity in unconfirmed TB. There are three problems with using presumptive-TB prevalence to estimate NAAT sensitivity:**
   a. **They (rightly, for their model structure) define their “non-infectious” TB state as asymptomatic, which presumptive TB patients are not.**
   b. **Presumptive-TB patients include many people with TB and higher bacillary burdens than the noninfectious-TB population.**
   c. **More fundamentally, there just isn’t any direct relationship between the prevalence of NAAT positivity among presumptive-TB patients (a mix of people with symptomatic bacteriologically positive TB, symptomatic bacteriologically negative TB, and no TB) and the sensitivity (prevalence of NAAT positivity) among people with [symptomatic] bacteriologically negative TB.**

**If there are no better data that can be used to estimate this parameter, then that high uncertainty should be made more explicit and explored more fully in sensitivity analyses.**

We thank the reviewer for picking this up. We recognise that this is an inconsistency in the manuscript. The issue lies in our wording of the definition rather than in the modelling assumptions themselves. The non-infectious TB state in our model is defined by the absence of bacteriological evidence and infectiousness, *regardless of symptoms*. This aligns with the “minimal TB” definition used in prior work (Horton et al., *Proc Natl Acad Sci* 2023), which similarly defines this state without conditioning on symptoms. We have now revised the manuscript to ensure consistency with this conceptual definition: “*non-infectious TB, representing individuals with inflammatory pathology in the absence of bacteriological evidence of TB disease and infectiousness, regardless of symptoms*” [Methods, lines 106-107; and S1 Text]. This definition is also consistent with the ICE-TB framework and current WHO definitions. We apologise for the oversight in our earlier wording. Furthermore, we note that the assumed NAAT test positivity values for non-infectious TB are similar to those used for individuals in the recovered state, which we believe mitigates concerns of overestimation regarding the impact of NAAT-based screening in this population.

7. **I would still like to see a sensitivity analysis related to assumptions of the underlying natural history model. I understand how the underlying state-transition parameters were estimated in previous work and how the screening intervention was applied simultaneously across states (and I apologize if my previous comment was not worded clearly), but I would like to see the sensitivity of the impact of repeated screening to the values of the transition rate from infected to noninfectious-TB, and to the proportion of TB that passes through the noninfectious state.**

We thank the reviewer for clarifying their earlier comment and for raising this thoughtful point. However, changing natural history parameters during an intervention period is not something usually done in screening models, in TB in general, or other disease models, as far as we have seen. While a very interesting line of enquiry, this is more a separate paper in itself, and beyond the scope of a sensitivity analysis in our paper at this stage, if only for the lack of data to inform such changes in transitions between parameters.

Please let us know if we can provide any additional information. We look forward to hearing from you at your earliest convenience.

Warm wishes,

Alvaro Schwalb, for the authors
